# Atomic-scale observation of solvent reorganization influencing photoinduced structural dynamics in a copper complex photosensitizer†

**DOI:** 10.1039/d2sc06600a

**Published:** 2023-02-01

**Authors:** Tetsuo Katayama, Tae-Kyu Choi, Dmitry Khakhulin, Asmus O. Dohn, Christopher J. Milne, György Vankó, Zoltán Németh, Frederico A. Lima, Jakub Szlachetko, Tokushi Sato, Shunsuke Nozawa, Shin-ichi Adachi, Makina Yabashi, Thomas J. Penfold, Wojciech Gawelda, Gianluca Levi

**Affiliations:** a Japan Synchrotron Radiation Research Institute Kouto 1-1-1, Sayo Hyogo 679-5198 Japan; b RIKEN SPring-8 Center 1-1-1 Kouto, Sayo Hyogo 679-5148 Japan tetsuo@spring8.or.jp; c XFEL Division, Pohang Accelerator Laboratory Jigok-ro 127-80 Pohang 37673 Republic of Korea; d European XFEL Holzkoppel 4, Schenefeld 22869 Germany; e Science Institute, University of Iceland 107 Reykjavík Iceland giale@hi.is; f DTU Physics, Technical University of Denmark Kongens Lyngby Denmark; g Wigner Research Centre for Physics, Hungarian Academy of Sciences H-1525 Budapest Hungary; h SOLARIS National Synchrotron Radiation Centre, Jagiellonian University PL-30392 Kraków Poland; i Institute of Materials Structure Science, High Energy Accelerator Research Organization (KEK) 1-1 Oho Tsukuba Ibaraki 305-0801 Japan; j Department of Materials Structure Science, School of High Energy Accelerator Science, The Graduate University for Advanced Studies 1-1 Oho Tsukuba Ibaraki 305-0801 Japan; k Chemistry-School of Natural and Environmental Sciences, Newcastle University Newcastle Upon-Tyne NE1 7RU UK; l Departamento de Química, Universidad Autónoma de Madrid, Campus Cantoblanco 28047 Madrid Spain; m IMDEA-Nanociencia, Campus Cantoblanco C/Faraday 9 28049 Madrid Spain; n Faculty of Physics, Adam Mickiewicz University 61-614 Poznań Poland

## Abstract

Photochemical reactions in solution are governed by a complex interplay between transient intramolecular electronic and nuclear structural changes and accompanying solvent rearrangements. State-of-the-art time-resolved X-ray solution scattering has emerged in the last decade as a powerful technique to observe solute and solvent motions in real time. However, disentangling solute and solvent dynamics and how they mutually influence each other remains challenging. Here, we simultaneously measure femtosecond X-ray emission and scattering to track both the intramolecular and solvation structural dynamics following photoexcitation of a solvated copper photosensitizer. Quantitative analysis assisted by molecular dynamics simulations reveals a two-step ligand flattening strongly coupled to the solvent reorganization, which conventional optical methods could not discern. First, a ballistic flattening triggers coherent motions of surrounding acetonitrile molecules. In turn, the approach of acetonitrile molecules to the copper atom mediates the decay of intramolecular coherent vibrations and induces a further ligand flattening. These direct structural insights reveal that photoinduced solute and solvent motions can be intimately intertwined, explaining how the key initial steps of light harvesting are affected by the solvent on the atomic time and length scale. Ultimately, this work takes a step forward in understanding the microscopic mechanisms of the bidirectional influence between transient solvent reorganization and photoinduced solute structural dynamics.

## Abstract 2

Ultrafast X-ray measurements reveal the mechanisms of the solvent influence on photosensitizing activity.

## Graphical abstract

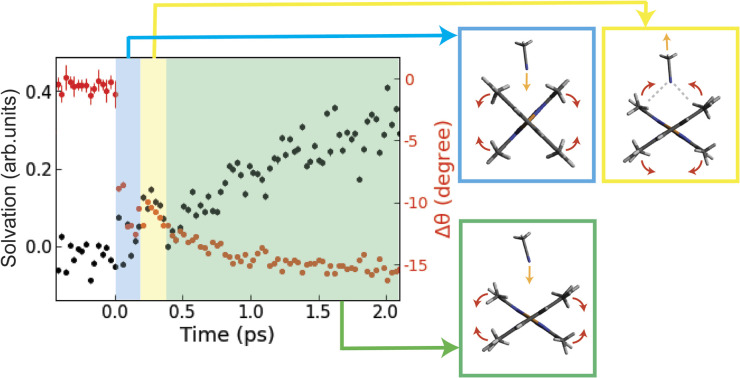

## Introduction

Solvation dynamics are ubiquitous and often crucial to understanding photoreactions across chemistry and biology. Transient atomic rearrangements in the solvation shell in response to electronic and nuclear structural changes of a photoexcited solute significantly impact the energetics of the excited states through specific solute–solvent interactions. Indeed, the reactant and product potential energy landscapes and energy crossings are modified by the solvent. As a result, the solvent can act as an energy source for promoting a reaction or as a heat bath for dissipating the excess excitation energy to stabilize intermediates. This leads to a greater diversity^1–3^ of reaction pathways, rates, products, and photophysical properties of excited species by a combination of solute and solvent molecules compared to gas-phase photochemistry. Time-resolved optical spectroscopy techniques have long been applied to characterize the rates of chemical reactions in different solvents.^1^ However, while these methods are sensitive to changes in solvation free energy, they do not provide direct information on specific short-range atomic rearrangements. Consequently, the transient solvent response and coupling to the solute dynamics on the atomic length scale remain elusive from an experimental point of view, and it remains poorly understood how specific solute–solvent interactions influence chemical reactions in the condensed phase.

**Fig. 1:**
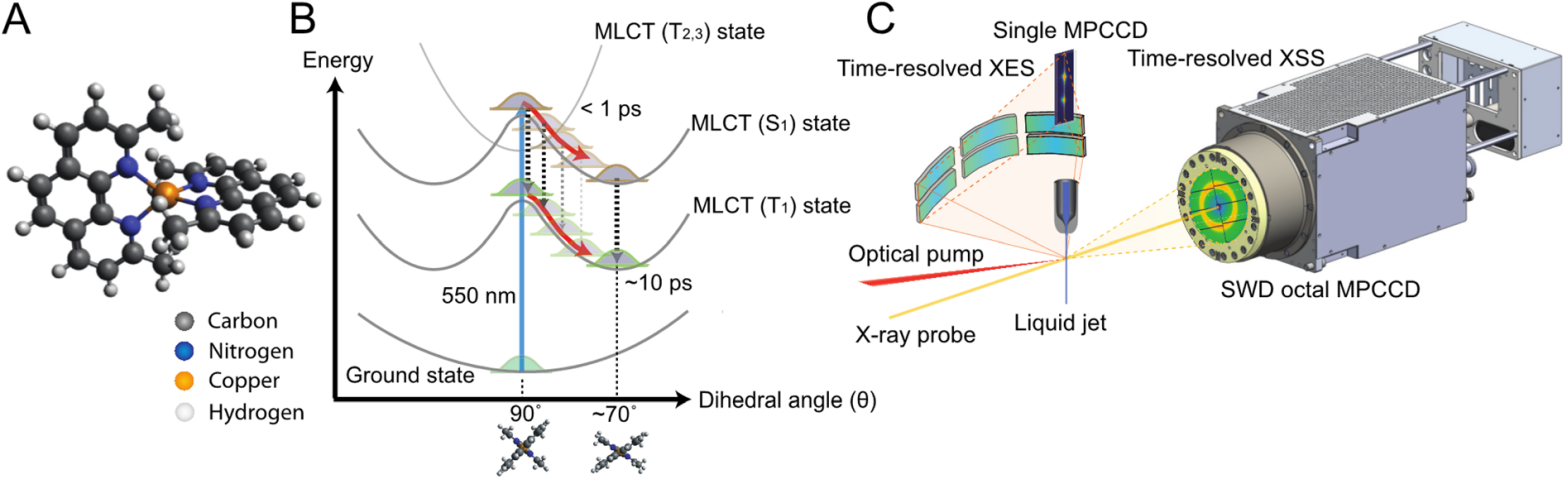
Schematics of [Cu(dmphen)_2_]^+^ and the experimental setup. (A) Ground state structure of [Cu(dmphen)_2_]^+^. (B) General picture of the potential energy surfaces of [Cu(dmphen)_2_]^+^. Within 1 ps, intersystem crossing (S_1_ → T_1_) *via* T_2_ and T_3_ states induces a branching of population between S_1_ and T_1_ states. After the flattening distortion is completed, intersystem crossing on a slower timescale (∼10 ps) proceeds from the S_1_ potential minimum. The geometries and the shape of the potentials of the S_1_ and T_1_ states are similar. (C) Instrumental layout enabling simultaneous time-resolved X-ray solution scattering and X-ray emission spectroscopy measurements.

These limitations are especially pertinent in the context of Cu(i) diimine transition metal complexes, *e.g.*, [Cu(dmphen)_2_]^+^ (dmphen = 2,9-dimethyl-1,10-phenanthroline, Fig. 1A), which has been studied^4–26^ both experimentally and theoretically as a prototype model of earth-abundant Cu(i) photosensitizers.^27–30^ The photosensitizing performance of this complex depends on the lifetime of the lowest triplet (T_1_) metal-to-ligand-charge-transfer (MLCT) state, which is strongly influenced by the solvent:^16^ it is over one order of magnitude shorter in electron donor solvents, such as acetonitrile (1.6 ns), than in nondonor solvents, such as dichloromethane (90 ns). The metastable T_1_ MLCT state is populated within ∼10 ps after visible light absorption and has a flattened structure associated with a pseudo-Jahn–Teller (PJT) distortion (Fig. 1B). It is generally understood that interactions with solvent molecules and the flattening distortion stabilize the T_1_ state, accelerating the decay to the ground (S_0_) state.^11,25^ While the general guiding principles for the rational design of Cu(i) photosensitizers are known (preventing solute–solvent interactions and suppressing the PJT distortion), the detailed atomic-scale mechanisms of the flattening distortion and interplay with the solvent response are still unclear, limiting the full exploitation of this class of complexes as photosensitizers. In the present work, we address the mechanism of the flattening distortion by directly exciting to the lowest singlet (S_1_) MLCT state. The flattening mechanism has been the object of previous different interpretations.^31^ Here, these controversies are resolved by providing a unified experimental and theoretical atomic-level mechanistic picture.

By using ultrafast optical emission spectroscopy, Iwamura *et al.*^9^ found that the time constant of the ligand flattening is longer in more viscous solvents, such as dichloromethane (700–900 fs), than in the less viscous solvent acetonitrile (340 fs). Ultrafast optical and X-ray absorption measurements^10,24^ also detected coherent nuclear vibrations arising predominantly from a breathing vibrational mode, which is activated upon photoexcitation owing to the contraction of the Cu–N bond lengths. The observed vibrational frequency (∼120 cm^−1^) is similar in dichloromethane and acetonitrile, while decoherence occurs on the same timescale as the flattening and is thus solvent-dependent. Different mechanisms of the flattening on the S_1_ and T_1_ surfaces have been proposed in previous studies.^31^ On one hand, an early ultrafast spectroscopic study by Chen and co-workers^8^ suggests that the flattening is fast and driven by spontaneous PJT instability. This view is also supported by the quantum mechanics/molecular mechanics (QM/MM) MD simulations in acetonitrile of ref. 25 and used in the present work. On the other hand, Tahara and co-workers^9,10^ interpreted the correlation between the lifetime of vibrational coherence and flattening obtained in ultrafast spectroscopic measurements in solution as an indication that vibrational coherence is maintained for a finite time in the S_1_ state before the MLCT states undergo the flattening distortion. Accordingly, they propose the presence of a shallow energy barrier between the perpendicular and flattened geometries, in contradiction with the typical spontaneous instability of PJT effects.^15,31–33^ However, an estimate of the minimum energy path between these two structures in the excited state using density functional theory (DFT) calculations presented in ref. 25 could not find an energy barrier. This controversy is still unresolved because none of the previous experimental studies could directly probe the flattening dynamics on the S_1_ and T_1_ surfaces with atomic structural sensitivity. Another controversy regards the formation of a penta-coordinated exciplex, which is discussed in a large body of literature.^4–26^ While early studies^4–8,12,16^ proposed and supported the exciplex formation, recent studies^11,24,25^ excluded this scenario and attributed the solvent dependency of the lifetime of the T_1_ state purely to intermolecular nonbonded interactions between solute and solvent molecules. One important limitation of previous experimental studies is that they could not capture the transient atomic rearrangements in the solvation shell after photoexcitation. Consequently, the atomic-level mechanisms that govern the influence of the solvent on the ligand flattening dynamics and vibrational coherence, thus modulating the lifetime of the T_1_ state, remain unclear.

X-ray free-electron lasers (XFELs) generate unprecedentedly brilliant, coherent, and ultrashort X-ray pulses, which allow the tracking of motions of electrons and nuclei in solution with sub-angstrom and femtosecond spatiotemporal resolutions. In particular, time-resolved X-ray solution scattering (XSS) at XFELs directly probes photoinduced changes in the distribution of atom pair distances. This unique capability, often combined with MD simulations, has enabled quantitative measurements of not only key motions of solute molecules but also the concomitant reorganization of the first solvation shell in various systems,^34–37^ ranging from small transition metal complexes^38–47^ to biological macromolecules.^48^ The ability of XSS to track transient structural dynamics in the solute and in the solvent has been demonstrated in previous studies on solvated metal complexes. For example, van Driel *et al.*^40^ could characterize the metal–metal contraction and ligand twisting in a diiridium complex as well as two distinct solvation processes (rotation and translation) of acetonitrile molecules around the Ir sites after photoexcitation. More recently, Biasin *et al.*^47^ observed transient translational motions of water molecules following photoinduced charge transfer excitation in a cyano-bridged bimetallic complex. Both studies shed light on key solute and solvent dynamics that are elusive to access with traditional optical probing. However, although previous studies^40,47^ tracked the solvation dynamics induced by electronic and structural changes in solute molecules, the influence of the transient solvent reorganization on the solute dynamics has so far eluded experimental determination. Generally, solute and solvent motions are entangled and mutually influence each other in photochemical processes in solution. As a consequence, a key challenge remains to fully elucidate the microscopic mechanisms governing the intertwined solute and solvent motions. These processes have been suggested to regulate the efficiency of Cu(i) photosensitizers, but have not been fully clarified yet. In this work, we address this issue by scrutinizing for the first time at the atomic level unclear aspects of the ligand flattening dynamics of solvated [Cu(dmphen)_2_]^+^.

Herein, we use time-resolved XSS and X-ray emission spectroscopy (XES) in combination with QM/MM MD simulations to elucidate the structural dynamics of [Cu(dmphen)_2_]^+^ (Fig. 1A) upon 550 nm photoexcitation in acetonitrile. At this optical wavelength, the S_0_ state is prevalently excited to the S_1_ MLCT state. The transient XES signal in the Cu Kα region (corresponding to 2p → 1s transitions) reflects changes in the local oxidation and spin state of the central Cu atom; thus, it is used to determine the population kinetics of the MLCT states. Typically, a strong correlation is found between an excitation fraction and the magnitude of structural changes in the XSS analysis, hampering a reliable structural determination. Since the time-resolved XES and XSS signals were recorded simultaneously (Fig. 1C), the time-dependent fraction of molecules in MLCT states is identical in both measurements. Hence, the population kinetics extracted from the XES data is used to constrain the XSS analysis and reduce the number of coupled fitting parameters, enabling a robust and reliable structural determination. The XSS analysis is further aided by the QM/MM MD simulations, which model changes in solute–solvent atom pair distances after photoexcitation. The solute–solvent radial distribution functions are used to model the scattering signal arising from changes in solute–solvent distances for the analysis of the XSS data, while the flattening dynamics from the nonequilibrium evolution of the excited state trajectories is directly compared to the results of the structural fitting. The interpretation of the experimental signal guided by the simulations provides an atomic-level mechanistic picture of the flattening dynamics and how it is modulated by the solvent. The obtained results unambiguously show that the ligand flattening proceeds in two steps. In the first step, sudden flattening of the phenanthroline ligands driven by PJT instability induces coherent vibrations along the flattening coordinate. This first step is independent of the solvent. In contrast, the second step is modulated by the solvent reorganization. The sudden flattening in the first step leads to coherent motions of acetonitrile molecules in the first coordination shell, which transiently approach and then move away from the metal. In turn, this solvent response induces loss of vibrational coherence along the flattening coordinate within a single oscillatory period. This is followed by the slower approach of acetonitrile molecules and further ligand flattening on a ∼500 fs timescale. Coherent vibrations along the breathing mode decay on a similar timescale as the second flattening process. These results enable us to draw a comprehensive picture of entangled solute and solvent structural dynamics. Ultimately, the combination of time-resolved XES, XSS, and QM/MM MD simulations allows us to unravel the interplay between solvent reorganization, ligand flattening and coherent vibrations of [Cu(dmphen)_2_]^+^ in acetonitrile at the atomic level, advancing the mechanistic understanding of the photoinduced dynamics of solvated Cu(i) phenanthroline photosensitizers.

## Results and discussion

### MLCT population kinetics from Cu Kα XES analysis

**Fig. 2:**
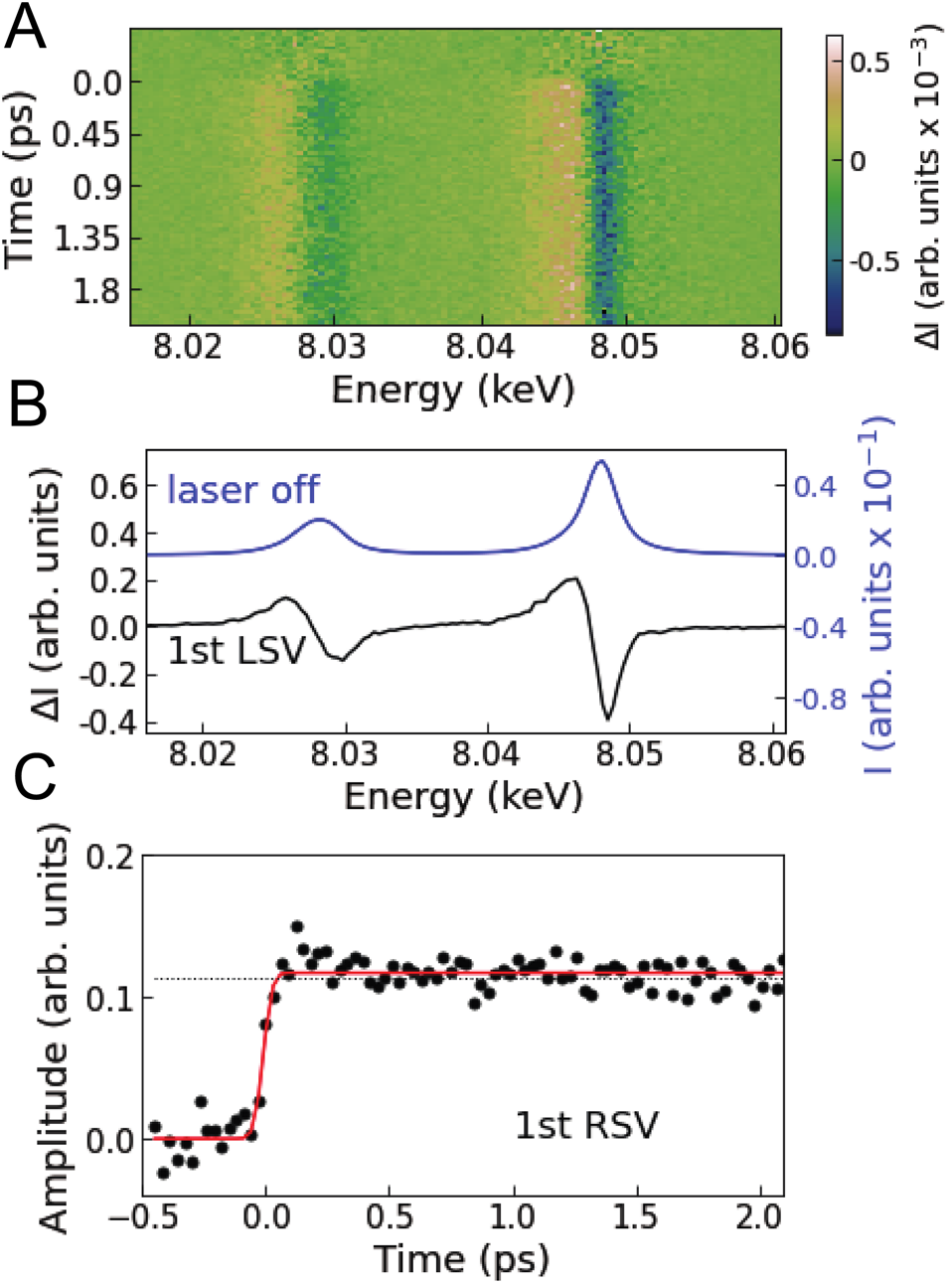
Time-resolved Cu Kα XES. (A) Measured difference Cu Kα XES spectra at various time delays. (B) Black line is the first left singular vector (1st LSV) of an SVD of the time-resolved XES data. The blue line is the static Cu Kα XES spectrum. (C) Black circles are the first right singular vector (1st RSV) of the SVD. The black dotted line corresponds to the amplitude of the 1st RSV at 18.8 ps. The red line is a fitted step function convoluted with a Gaussian instrument response function.

Fig. 2A shows the Cu Kα XES difference spectra of [Cu(dmphen)_2_]^+^ in acetonitrile measured at various time delays. Singular value decomposition (SVD) identifies only one time-dependent, distinct singular vector, implying that only one spectral component is sufficient to account for the changes in the Kα XES signal (Note S1 and Fig. S1 in the ESI†). The corresponding first left singular vector (Fig. 2B; 1st LSV) can be explained by a red energy shift of the static spectrum. The Kα spectral shape of 3d transition metals is mainly correlated with the number of unpaired 3d electrons through 3d–2p exchange interactions.^49–51^ Accordingly, the change in the Cu Kα XES spectrum is associated with the total fraction of molecules in MLCT states after photoexcitation (Cu^1+^: d^10^ → Cu^2+^: d^9^) and is insensitive to internal conversion or intersystem crossing within the MLCT manifold. This is because the latter processes correspond to the spin flip of the excited electron located in a molecular orbital localized on the ligands and thus do not modify the oxidation or spin state of the central Cu ion. Consequently, the first right singular vector (Fig. 2C; 1st RSV), *i.e.*, the time-dependent amplitude of the 1st LSV, reflects the total MLCT yield. In Fig. 2C, the signal (black circles) is overlaid with a fitted step function broadened with a Gaussian instrument response function (IRF; Table S1 in the ESI†), showing no clear decay up to the maximum time delay covered in the experiment (18.8 ps). This means that the fraction of photoexcited molecules that decay to the ground state (S_i_ → S_0_ or T_i_ → S_0_) is negligibly small within the experimental time window, which is consistent with estimates based on the absolute radiative rate constants^9^ of the MLCT states (Note S1 in the ESI†). Therefore, it is reasonable to approximate the total MLCT population as time-independent within the range of time delays of the measurement. In the following analysis and interpretation of the time-resolved XSS data, the MLCT population is fixed at 20.2%, as determined by scaling a reference spectrum corresponding to 100% excitation to the transient spectrum averaged over time intervals above 0.5 ps (Note S1 and Fig. S2 in the ESI†).

### Ultrafast structural dynamics revealed by time-resolved XSS

**Fig. 3:**
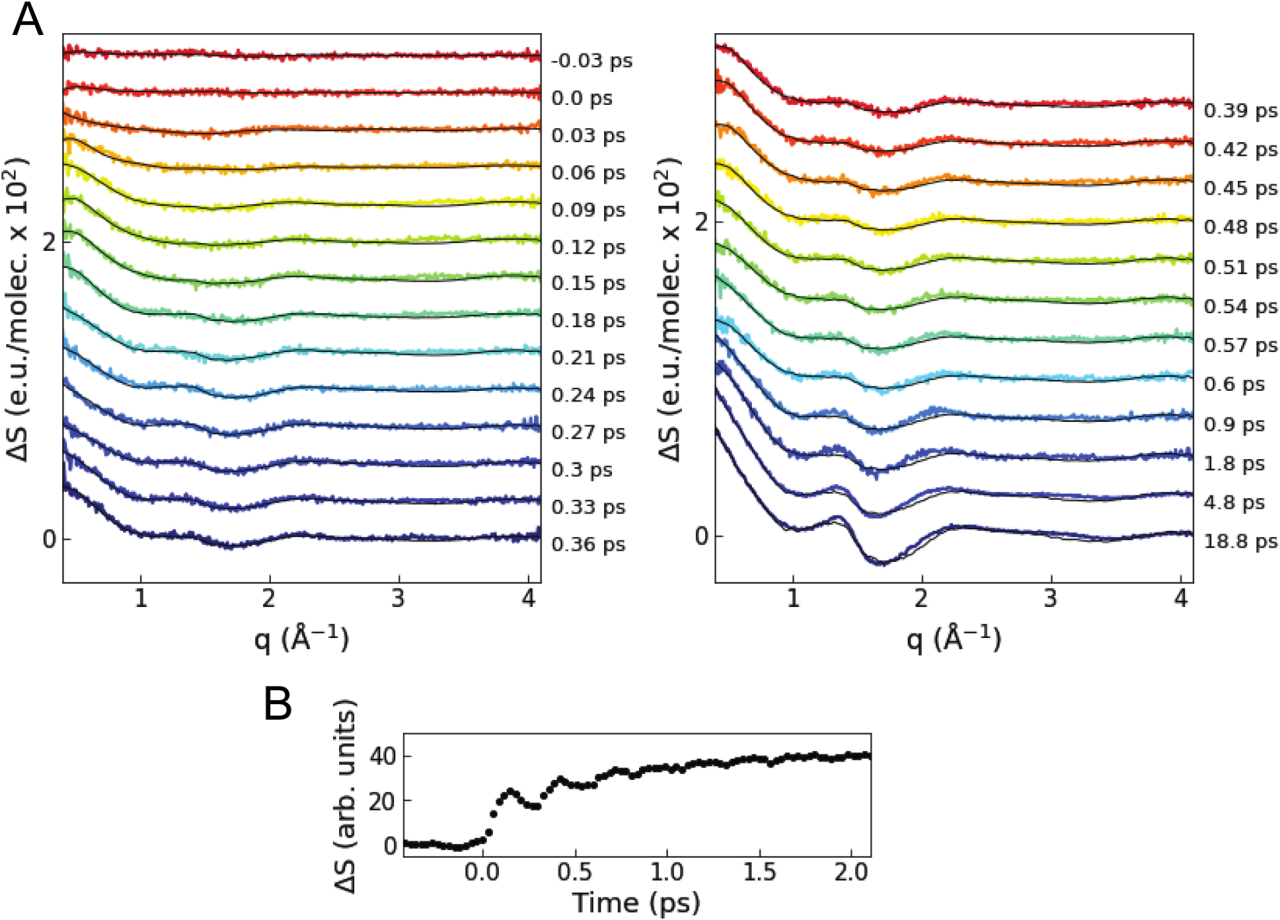
Time-resolved XSS signal. (A) Colored lines are the difference scattering curves recorded at various time delays (vertically offset for clarity). The overlaid black lines correspond to the fitted curves of the quantitative structural analysis. (B) Time evolution of the intensity of the scattering signal averaged in the low *q* range of 0.4–0.8 Å^−1^.

Fig. 3A shows the isotropic difference scattering signal Δ*S*(*q*,*t*) as a function of time delay *t* and length of the scattering vector ***q***. After time zero, a strong positive feature is observed in the low *q* region (0.4–0.8 Å^−1^), which indicates an average decrease in interatomic distances upon excitation. In the first 1 ps, the average signal in this low *q* region shows oscillations with a period of ∼280 fs superimposed on an exponential growth (Fig. 3B). The close similarity with the period of the breathing mode observed in previous ultrafast optical and X-ray absorption measurements^10,24^ suggests that these changes in the XSS signal reflect coherent oscillations and contraction of the Cu–N bond lengths in the MLCT states, as confirmed by the quantitative analysis described below. The signal in the 1.5–2.0 Å^−1^*q* region has a shape similar to the difference scattering signal of bulk acetonitrile^52^ when the temperature is increased at a constant volume (Note S2 and Fig. S3 in the ESI†).

We perform a standard quantitative analysis,^53–58^ where Δ*S*(*q*,*t*) is modelled as a sum of three difference scattering components reflecting changes in the solute–solute (Δ*S*^solute–solute^), solute–solvent (Δ*S*^solute–solvent^), and solvent–solvent (Δ*S*^solvent–solvent^) atom pair distances:

1 Δ*S*_model_(*q*,*t*) = Δ*S*^solute–solute^(*q*,*t*) + Δ*S*^solute–solvent^(*q*,*t*) + Δ*S*^solvent–solvent^(*q*,*t*)

The model includes four time-dependent parameters (Note S2 in the ESI†). In modelling Δ*S*^solute–solute^, an ensemble of MLCT structures is approximated as a single representative structure. Furthermore, the scattering signals arising from the singlet and triplet MLCT states are assumed to be indistinguishable, *i.e.*, intersystem crossing does not affect the structural dynamics. This assumption is justified owing to the known close similarities between the S_1_ and T_1_ potential energy surfaces and geometries.^14,24^ A simulated solute–solute signal Δ*S*^solute–solute^_sim_ is obtained from representative MLCT structures generated by varying the average Cu–N bond length *r*(*t*) and the NNCuNN dihedral angle *θ*(*t*) from the S_0_ structure optimized with density functional theory (more details are available in the Methods and Note S2 in the ESI†). Hence, Δ*S*^solute–solute^_sim_ incorporates the change in the average Cu–N bond length Δ*r*(*t*) and the change in the interligand dihedral angle Δ*θ*(*t*) as time-dependent parameters. A solute–solvent signal Δ*S*^solute–solvent^_sim_ is calculated^59^ using equilibrium solute–solvent radial distribution functions from the QM/MM MD simulations^25^ and multiplied by a scaling factor *α*(*t*) to model Δ*S*^solute–solvent^ (Note S2 and Fig. S4–S7 in the ESI†). The use of a single solute–solvent component calculated using equilibrium solute–solvent radial distribution functions is justified by the fact that an SVD analysis of the time-dependent difference scattering signal calculated from the nonequilibrium excited state trajectories is dominated by a single component with a shape very similar to the signal from equilibrium radial distribution functions (see Fig. S4 in the ESI†). Fig. S5 in the ESI† shows that changes in the Cu–N bond length, dihedral angle and solvation structure are associated with significantly different scattering signals, enabling a robust structural determination through fitting without additional assumptions about the correlation between these parameters. The bulk solvent component Δ*S*^solvent–solvent^ is approximated by multiplying the change in solvent temperature Δ*T*(*t*) by a reference difference scattering curve ∂(Δ*S*^solvent^_ref_(*q*))/∂*T* separately recorded using a dye solution (Note S2 and Fig. S3 in the ESI†). The MLCT fraction *A*_MLCT_ is fixed at 0.202 on the basis of the XES analysis. The resultant fitting model is:

2 Δ*S*_model_(*q*,*t*) = *A*_MLCT_·{Δ*S*^solute–solute^_sim_(Δ*θ*(*t*),Δ*r*(*t*)) + *α*(*t*)·Δ*S*^solute–solvent^_sim_(*q*)} + Δ*T*(*t*)·∂(Δ*S*^solvent^_ref_(*q*))/∂*T*

Δ*S*_model_ is fitted to the data at each time delay using a maximum likelihood estimation (Fig. S8–S10 and Tables S2 and S3 in the ESI†).

**Fig. 4:**
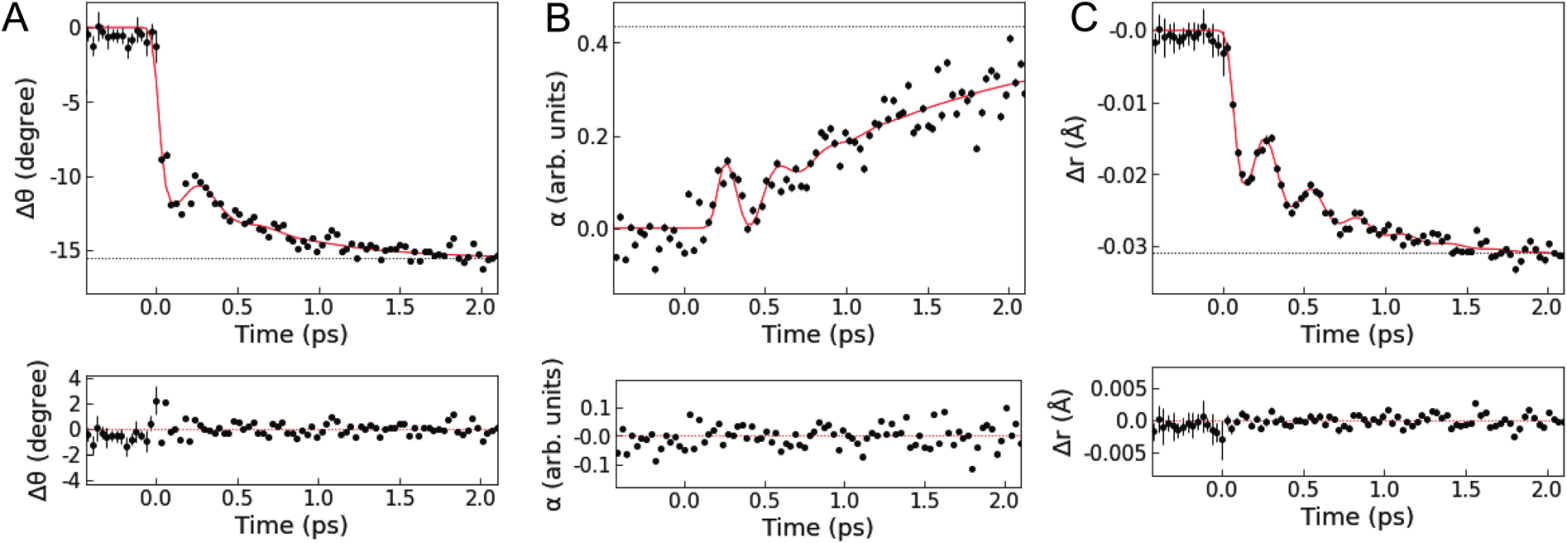
Intramolecular and solvation structural dynamics. (top, A) Change in the NNCuNN dihedral angle Δ*θ*(*t*). (top, B) Scaling factor of the solute–solvent component *α*(*t*). (top, C) Change in the average Cu–N bond length Δ*r*(*t*). The black circles correspond to the values obtained from the structural analysis of the XSS data. Black dotted lines correspond to the values at 18.8 ps. The red lines are kinetic fitting curves. (bottom, A–C) Residuals of the kinetic fitting.

Fig. 4 shows the results of the quantitative analysis of the time-resolved difference scattering signal. After photoexcitation, an instantaneous decrease in the dihedral angle *θ*(*t*) faster than the IRF width is observed, followed by coherent oscillations. The oscillations are damped after only one period, followed by a further exponential decrease in *θ*(*t*). Δ*θ*(*t*) is fitted with the sum of a step, an exponential, and a damped sine function convoluted with the IRF (the red line in Fig. 4A, see also Note S3 and Table S3 in the ESI†), giving a time constant of 554 ± 67 fs for the exponential decrease. The fit gives a period of oscillations along the flattening coordinate of 401 ± 29 fs. This period is shorter than the period obtained from a normal mode analysis with the optimized geometry of the T_1_ or S_1_ states (∼925 fs, see Fig. S21 in the ESI†) using DFT calculations in a vacuum or with solvent effects included implicitly through the conductor-like polarizable continuum model (CPCM). This discrepancy indicates that the motion along the flattening coordinate is influenced by specific solute–solvent interactions beyond the CPCM model, highlighting the importance to directly probe intertwined solute and solvent motions on the atomic length scale. Except for the rapid initial change, similar trends are observed in the scaling factor of the solvation component *α*(*t*) (strongly damped oscillations followed by an exponential rise, see Fig. 4B), which is fitted with the sum of an exponential and a damped sine function. The oscillations in *α*(*t*) have the same period as the oscillations in Δ*θ*(*t*) but have a phase lag with respect to them (Note S3 and Fig. S11 in the ESI†). These characteristics indicate that the ligand flattening dynamics is coupled to the solvent reorganization.

In Fig. 4C, Δ*r*(*t*) shows a rapid decrease followed by an exponential decrease superimposed with an oscillatory signal associated with the breathing motion. The observed Δ*r*(*t*) is fitted with the same model as employed for the fitting of Δ*θ*(*t*). The fit gives a period of 286 ± 5 fs (∼117 cm^−1^) and damping time of 350 ± 57 fs for the coherent oscillations, an exponential decay time constant of 582 ± 41 fs, and a saturated Cu–N bond length contraction of −0.032 Å. The analysis of the evolution of the mean Cu–N distance from the nonequilibrium QM/MM MD trajectories (see Fig. S22 in the ESI†) gives a main vibrational period of ∼282 fs and a contraction of ∼−0.035 Å in very good agreement with the results of the fit. A vibrational analysis with the S_1_ geometry optimized with DFT calculations in a vacuum gives a breathing normal mode with a period of 290 fs (see Note S3 and Fig. S21 in the ESI†), further confirming the assignment of these coherent oscillations to the Cu–N breathing and showing that the vibrational frequency of this mode is only slightly affected by the solvent. These results are also in excellent agreement with previous studies^6,10,11,24^ reporting the Cu–N breathing vibrational mode and the magnitude of the shortening of the Cu–N bond lengths in the relaxed T_1_ state. There are two possible scenarios to account for the exponential decrease in *r*(*t*) underlying the coherent oscillations. One is that anharmonicity leads to an effective exponential decrease of the ensemble average Cu–N distances as the wave packet traverses the Cu–N potential. The other is that the local potential minimum along the Cu–N coordinate shifts toward shorter Cu–N distances as the dihedral angle *θ*(*t*) decreases. Since the QM/MM MD simulations^25^ found anharmonic effects to be small, the latter appears to be the most likely scenario. This is further supported by the fact that the exponential decreases in *θ*(*t*) and *r*(*t*) show the same time constant (∼550 fs) within the uncertainty defined by the standard errors.

**Fig. 5:**
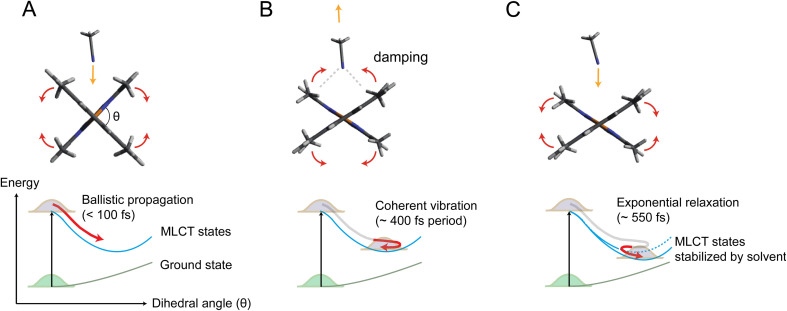
Mechanism of coupled ligand flattening and solvation dynamics deduced from the XSS analysis. (A) Initially, the propagation of the nuclear wave packet along the potential energy landscape is ballistic and flattening of the ligands occurs in less than 100 fs. Acetonitrile molecules approach the Cu atom by intercalating through the flattened ligands. (B) Acetonitrile molecules coherently rebound as the ligands slightly move again towards the perpendicular geometry. Enhanced solute–solvent interactions lead to damping of coherent vibrations along the flattening coordinate. (C) The incoming acetonitrile molecules stabilize the flattened MLCT geometry inducing a further ligand flattening on slower timescales, which, in turn, facilitates the further approach of solvent molecules to the Cu atom.

Fig. 5 illustrates the mechanism of the ligand flattening dynamics deduced from the observed time evolutions of Δ*θ*(*t*) and *α*(*t*). The prompt decrease in the dihedral angle *θ*(*t*) followed by the oscillations in the early timescales can be interpreted as ballistic propagation of the nuclear wave packet along the flattening coordinate representing the PJT distortion, which leads to structural trapping and initiates coherent vibrations around the potential minimum. This fast initial dynamics is driven by instantaneous PJT instability and thus, is an intrinsic process of [Cu(dmphen)_2_]^+^, largely independent of the presence of surrounding solvent molecules. This observation confirms that PJT distortion is spontaneous in [Cu(dmphen)_2_]^+^, which is expected based on the electronic structure of d^9^ complexes but has previously been questioned by Iwamura *et al.*^9,10^ Here, we solve this ambiguity by directly observing and characterizing experimentally the flattening distortion for the first time compared to previous studies.^4–25^

The following oscillations in *α*(*t*) in the early timescales reflect the solvent response to the change in *θ*(*t*). The QM/MM MD-simulated solute–solvent radial distribution functions used to calculate the solvation signal Δ*S*^solute–solvent^_sim_ are dominated by a shortening of the distances between the Cu and atoms (N and C) of the closest solvent molecules in the excited state compared to the ground state (Note S4, Fig. S13 and S14 in the ESI†). Therefore, the oscillations in *α*(*t*) correspond to the collective motions of acetonitrile molecules approaching and subsequently departing from the central Cu atom; they first intercalate through the flattened ligands and then extrude away as *θ*(*t*) increases again in the coherent oscillations. The approach of acetonitrile molecules to the metal center leads to a strengthening of the solute–solvent interactions. It is, then, conceivable that the vibrational energy along the flattening coordinate is efficiently dissipated into the first solvation shell through the enhanced solute–solvent interactions, causing the strong damping of the coherent oscillations within a single oscillatory period observed in Fig. 4. Subsequently, the magnitude of both Δ*θ*(*t*) and *α*(*t*) shows an exponential growth, revealing a further interplay between solute and solvent molecules. The magnitude of *α*(*t*) saturates at ∼0.4 after the exponential growth (Table S3 in the ESI†). According to the excited state QM/MM MD simulations, acetonitrile molecules approach the Cu atom only up to 3 to 4.75 Å (ref. 25) (distance between the Cu atom and the N atom of the solvent). Altogether, these results show that the intercalated acetonitrile molecules interact weakly with the metal atom and do not form penta-coordinated exciplex species, corroborating the observations in our previous studies.^11,24^ The electrostatic nonbonded interactions involving incoming acetonitrile molecules cause a stabilization of the MLCT states with the flattened structure and, hence, a further approach of solvent molecules to the metal. Although these solute–solvent interactions are weak compared to the strength of the chemical bond, they are sufficient to modify the shape and position of the potential energy surface.^11,25^ The solute–solvent interactions mediating the flattening distortion could also promote the damping of other vibrations, *e.g.*, the breathing motion, and, thus, regulate the lifetime of vibrational coherence. This damping pathway can explain why the flattening distortion and decoherence of vibrational modes occur on similar timescales. The solvent influence on vibrational decoherence, together with the observation that the flattening is initially fast and spontaneous, excludes the previous interpretation by Iwamura *et al.*,^9,10^ which assumes the presence of an energy barrier between the perpendicular and planar structures, while it confirms the early observation by Shaw *et al.*^8^ and the result of the QM/MM MD simulations of ref. 25 that the flattening is driven by spontaneous PJT instability. In previous experimental studies,^6,8–10,12,18,19,22–24^ the flattening distortion was interpreted using a single time constant. In the present study, the flattening distortion is clearly observed to occur in two distinct steps; the first is fast and driven by spontaneous PJT instability, while the second is slow and modulated by the solvent. This biphasic ligand flattening dynamics in acetonitrile is further confirmed by the analysis of the nonequilibrium part of the QM/MM MD trajectories.^25^ Fig. S12 in the ESI† shows a comparison between the change in the dihedral angle extracted from the XSS analysis and an exponential fit of the evolution of the dihedral angle from the QM/MM MD trajectories. A good agreement in the flattening dynamics between the experiment and simulations is observed. As demonstrated in ref. 25, two time constants are needed to adequately describe the evolution of the ligand flattening in the simulations, a short (110 fs) and a long (1.2 ps) time constant, corresponding respectively to the initial fast and slower solvent-modulated flattening steps also observed in the XSS measurements. On the other hand, the QM/MM MD simulations overestimate the damping of coherent oscillations along the flattening coordinate. Therefore, the coherent oscillations in the solute flattening and solvation structural dynamics could only be uncovered here thanks to the experiments. This shows that further advancements in the simulations are needed to more accurately describe the complexity of solute–solvent interactions in the excited state dynamics, for example through the explicit quantum mechanical description of the solvent.

The mechanism presented here offers for the first time an experimentally deduced atomic-scale perspective to understand the influence of a donor solvent on the relaxation process of [Cu(dmphen)_2_]^+^, which leads to quenching of visible light emission,^3^ and therefore, dominates the photosensitizing properties of this model Cu(i) phenanthroline complex.

## Conclusions

By exploiting the sensitivity of time-resolved XSS to changes of interatomic distances in solution in combination with time-resolved XES and QM/MM MD simulations, we unambiguously disentangle two different steps in the photoinduced ligand flattening of [Cu(dmphen)_2_]^+^ in acetonitrile, which could previously not be resolved through optical techniques. Shortly after photoexcitation (delays < ∼400 fs), the excited MLCT states undergo prompt flattening due to PJT instability, launching coherent nuclear vibrations. This process is intrinsic to [Cu(dmphen)_2_]^+^ and largely independent of surrounding solvent molecules. Concomitantly, acetonitrile molecules in the first coordination shell reorganize by coming closer to the open metal center and subsequently rebound as the ligands move towards the perpendicular geometry in the coherent oscillations. This coherent solvent motion is responsible for an efficient dissipation of vibrational energy. At later times (delays > ∼400 fs), the further approach of acetonitrile molecules to the Cu atom and enhanced ligand flattening proceed synergistically. The decoherence of breathing mode vibrations occurs on a similar timescale as the second slow flattening motion. The second step in the structural dynamics is strongly dependent on the properties of the solvent such as polarity, electron donating capacity, and diffusion, which explains the solvent dependency of the PJT distortion and lifetime of the photosensitizing MLCT states, observed in previous studies.

More generally, the findings in the present work demonstrate that understanding the mechanisms of photoinduced structural dynamics of photoactive solvated molecules requires experimental techniques sensitive to solvent rearrangements at the atomic level. Time-resolved X-ray scattering and spectroscopic techniques complemented by QM/MM MD overcome the limitations of conventional ultrafast optical methods by mapping three-dimensional structural changes in real time as nuclear wave packets traverse the potential energy landscape. This unique capability to directly visualize nuclear motions, including the solvation dynamics, has been exploited here to elucidate for the first time the microscopic mechanisms of the influence of transient solvent motions on the solute structural dynamics. Together with the observation of the solvent reorganization induced by photoexcitation of the solute, this study demonstrates that an intricate interplay between solute and solvent motions affects the photosensitizing ability of a copper complex. Therefore, this study paves the way to better understanding the solvent effect on photoreactions across chemistry and biology.

## Experimental section

### X-ray measurements

A pump-probe experiment was performed at the SPring-8 Angstrom Compact free-electron LAser (SACLA), using the SACLA Pump-probe INstrumEnt for Tracking Transient dynamics (SPINETT) platform dedicated to ultrafast chemistry.^60,61^ An XFEL beam with a central photon energy of 10 keV and a bandwidth of ∼50 eV was focused down to 2.4 (H) × 4.1 (V) μm^2^ at the full width at half maximum (FWHM) by using compound refractive lenses.^62^ The time-resolved XSS and XES data were simultaneously recorded using short-working-distance octal (SWD octal) and single multiport charge-coupled device (MPCCD) detectors,^63^ respectively. For XSS, the SWD octal MPCCD detector was set at 60.9 mm behind an interaction point, and it covered lengths of the scattering vector ***q*** in the range of 0.4–4.1 Å^−1^. For XES, six cylindrically bent (0.25 m radius) Si(531) crystal analyzers were aligned in the von Hamos geometry to diffract Cu Kα X-ray emissions on the single MPCCD detector. The Bragg angle ranged from 56.16° to 58.50°, corresponding to an energy range of 7.92–8.13 keV. The [Cu(dmphen)_2_]PF_6_ complex was dissolved in acetonitrile at a concentration of 100 mM. The solution was delivered to the interaction point as a cylindrical jet through a 50 μm-diameter injector in a closed-loop circulating apparatus. 550 nm laser pulses with a 45 fs FWHM duration were generated by a Ti:sapphire laser system coupled with an optical parametric amplifier (HE-TOPAS; Coherent), and were focused down to a 122 (H) × 174 (V) μm^2^ spot size (FWHM). This wavelength prevalently excites the photosensitizer from the S_0_ state to the S_1_ state. To determine the optimal pump laser intensity to achieve the highest MLCT population with minimal multiphoton processes, we conducted a power titration scan and selected a pulse energy of 15.9 μJ (Note S5, Fig. S17 and S18 in the ESI†). With this optical intensity, an excitation fraction of 20.2 ± 0.240% was achieved. The arrival timing between X-ray and optical pulses was recorded using timing diagnostics based on the X-ray beam splitting scheme.^64,65^ The temporal jitter is corrected for time delays of ≤2.5 ps, using an interval bin width of 30 fs. The overall experimental time resolution was 67 fs at the FWHM, as evaluated from an SVD analysis of the time-resolved Cu Kα difference XES spectra (Note S1 and Table S1 in the ESI†). An SVD analysis of the time-resolved XSS data confirms that four time-dependent parameters do not cause overfitting in the structural determination (Note S6, Fig. S19 and S20 in the ESI†). The details of data collection and reduction schemes are described in Note S0 in the ESI.†

The static reference Cu Kα X-ray emission spectra of the [Cu(dmphen)_2_]PF_6_ (Cu^1+^: d^10^) and [Cu(dmphen)_2_](PF_6_)_2_ (Cu^2+^: d^9^) complexes were recorded at the FXE instrument^66^ of the European XFEL.^67^ These two samples were dissolved in acetonitrile to a concentration of ∼30 mM. The solutions were flowed as cylindrical jets through a 100 μm-diameter injector at a speed of ∼60 m s^−1^. The XFEL beam with a central photon energy of 9.3 keV and an average pulse energy of ∼1 mJ was focused down to <10 μm^2^ FWHM. The spectra were recorded in the burst mode operation of the European XFEL, delivering 150 pulses per burst at a 0.564 MHz repetition rate with a burst repetition rate of 10 Hz. The Cu Kα X-ray emissions from the two samples were diffracted by six cylindrically segmented (0.5 m radius) Si(111) crystal analyzers in the von Hamos geometry (Bragg angle of 79.7°) and focused on a Jungfrau 1 M detector.

### Simulations

The difference scattering signal arising from changes in the structure of [Cu(dmphen)_2_]^+^ (Δ*S*^solute–solute^_sim_) is calculated through the Debye equation, using a fixed ground state structure and excited MLCT state structures parametrized in terms of the average Cu–N bond length and NNCuNN dihedral angle. The ground state structure corresponds to the S_0_ geometry optimized with DFT, while the MLCT structures were generated as follows. First, the T_1_ structure is optimized with time-dependent DFT (TD-DFT). Then, a set of structures is generated by linear interpolation of the atomic distances of the optimized S_0_ and T_1_ geometries, followed by linear interpolation of the Cartesian positions of the nearest structures from this set (more details in Note S2 in the ESI†). Finally, for each of these in-between S_0_–T_1_ structures, the atoms of the phenanthroline ligands are displaced along the vectors from the Cu atom to the centers of mass of the phenanthroline ligands. The DFT and TD-DFT calculations to obtain the optimized geometries were performed using the ORCA 5.0.1 program package^68,69^ with the PBE0 functional^70^ and the def2-SVP and def2-TZVP basis sets^71^ for C, N, and H atoms and for the Cu atom, respectively. Solvent effects were taken into account using the conductor-like polarizable continuum model with *ε* = 36.6 for acetonitrile.

The QM/MM MD simulations are described elsewhere.^25^ In short, the simulations include the [Cu(dmphen)_2_]^+^ complex and 468 acetonitrile molecules and are based on a fixed QM/MM partition. The QM part includes only the complex described with DFT using the BLYP functional^72^ and tzp (Cu) and dzp (all other atoms) basis sets of localized numerical atomic orbitals.^73^ While for the solvent in the MM part, a three-site point charge interaction potential^25,74^ is used. The DFT calculations are performed with the Grid-based Projector Augmented Wave (GPAW) software.^75,76^ The QM and MM parts are coupled through an electrostatic embedding model as implemented in the Atomic Simulation Environment.^77,78^ Since the MM model for acetonitrile uses point charges with a fixed magnitude, the QM/MM coupling does not describe the polarization of the solvent by the electron density of the QM solute. This polarization can give rise to a fast electronic solvent response after photoexcitation of the solute. On the other hand, for the time scales investigated here, the large structural change of the complex leads to a significant nuclear reorganization within the solvation shell, which is expected to dominate the solvent response. Previous ultrafast studies on transition metal complexes^40,47^ have shown that such nuclear rearrangements in the solvent can be described with sufficient accuracy using a non-polarizable solvent model. 48 trajectories are propagated in the S_1_ state within direct Born–Oppenheimer MD for at least 4 ps each with initial conditions obtained from a large set of configurations generated by propagating ground state trajectories in the canonical ensemble at 300 K. Fig. S15 in the ESI† shows that 48 trajectories are enough to robustly characterize the time-dependent ensemble of solute–solvent distances, because the changes in radial distribution functions at a given time when increasing the number of trajectories beyond ∼40 are much smaller than the differences between different times. The excited state calculations use a time-independent, variational method (ΔSCF approach) implemented in GPAW^79^. The excited state potential energy surfaces and vibrational frequencies of polyatomic molecules computed with this method have been found to have comparable or higher accuracy than those calculated with TD-DFT.^79–83^ Moreover, variational DFT calculations of excited states have been employed in several previous MD studies including explicit solvation effects, where they could successfully predict the photoinduced structural dynamics of organic molecules and metal complexes in solution.^79,84^ The difference scattering signal arising from changes in solute–solvent atom pair distances (Δ*S*^solute–solvent^_sim_) is calculated^59^ from the solute–solvent radial distribution functions of the ground and excited state trajectories, considering for the latter only times over 3 ps, at which point the trajectories are sufficiently equilibrated.

## Data availability

All relevant data and programs are available from the corresponding authors upon request.

## Author contributions

T. K., T.-K. C., T. J. P., W. G., C. J. M., G. V., F. A. L., Z. N., S. N., S. A., T. S., M. Y., J. S., and D. K. conceived the proposal and designed the experiments in this project. T. K., T.-K. C., W. G., C. J. M., G. V., Z. N., J. S., F. A. L. and D. K. performed the experiments. G. L. and A. O. D. performed the QM/MM MD simulations. T. K., T.-K. C., T. J. P., G. V., D. K., S. N., S. A., G. L., and A. O. D. analyzed and interpreted the measured data. T. K., T. J. P., and G. L. wrote the manuscript with discussion and input from all authors.

## Conflicts of interest

The authors declare no competing interests.

## Supplementary Material

SC-014-D2SC06600A-s001
