# Supplementary material for: Atomic-scale observation of solvent reorganization influencing photoinduced structural dynamics in a copper complex photosensitizer
Source: Chem Sci. 2023 Feb 1;14(10):2572–84. doi: 10.1039/d2sc06600a (PMC9993854; doi:10.1039/d2sc06600a)
Supplement: SC-014-D2SC06600A-s001 [file SC-014-D2SC06600A-s001.pdf]

Electronic Supplementary Material (ESI) for Chemical Science.
This journal is © The Royal Society of Chemistry 2023

Atomic-scale observation of solvent reorganization influencing photoinduced structural dynamics in a copper complex photosensitizer

Tetsuo Katayama,[*a,b] Tae-Kyu Choi,[c] Dmitry Khakhulin,[d] Asmus O. Dohn,[e,f] Christopher J. Milne,[d] György Vankó,[g] Zoltán Németh,[g] Frederico A. Lima,[d] Jakub Szlachetko,[h] Tokushi Sato,[d] Shunsuke Nozawa,[i,j] Shin-ichi Adachi,[i,j] Makina Yabashi,[b] Thomas J. Penfold,[k] Wojciech Gawelda,[l,m,n] and Gianluca Levi[*e]

[a] *Japan Synchrotron Radiation Research Institute, Kouto 1-1-1, Sayo, Hyogo 679-5198, Japan*

[b] *RIKEN SPring-8 Center, 1-1-1 Kouto, Sayo, Hyogo 679-5148, Japan*

[c] *XFEL division, Pohang Accelerator Laboratory, Jigok-ro 127-80, Pohang 37673, Republic of Korea*

[d] *European XFEL, Holzkoppel 4, 22869 Schenefeld, Germany*

[e] *Science Institute, University of Iceland, 107 Reykjavík, Iceland*

[f] *DTU Physics, Technical University of Denmark, Kongens Lyngby, Denmark*

[g] *Wigner Research Centre for Physics, Hungarian Academy of Sciences, H-1525 Budapest, Hungary*

[h] *SOLARIS National Synchrotron Radiation Centre, Jagiellonian University, PL-30392 Kraków, Poland*

[i] *Institute of Materials Structure Science, High Energy Accelerator Research Organization (KEK), 1-1 Oho, Tsukuba, Ibaraki 305-0801, Japan*

[j] *Department of Materials Structure Science, School of High Energy Accelerator Science, The Graduate University for Advanced Studies, 1-1 Oho, Tsukuba, Ibaraki 305-0801, Japan*

[k] *Chemistry-School of Natural and Environmental Sciences, Newcastle University, Newcastle Upon-Tyne, NE1 7RU, United Kingdom*

[l] *Departamento de Química, Universidad Autónoma de Madrid, Campus Cantoblanco, 28047 Madrid, Spain*

[m] *IMDEA-Nanociencia, c/Faraday 9, Campus Cantoblanco, 28049 Madrid, Spain*

[n] *Faculty of Physics, Adam Mickiewicz University, 61-614 Poznań, Poland*

*e-mail: tetsuo@spring8.or.jp, giale@hi.is

Table of Contents:

Note S0: Data collection and reduction of time-resolved XSS and XES

Note S1: SVD analysis of time-resolved Cu Kα XES difference spectra

Note S2: Structural analysis of time-resolved XSS difference signals

2-1. Simulations of $\Delta S^{solute-solute}$, $\Delta S^{solute-solvent}$, and $\Delta S^{solvent-solvent}$

2-2. Sensitivity of XSS to structural degrees of freedom

2-3. Structural fitting

2-4. Fitting summary

Note S3: Kinetic time constants from the XSS analysis

3-1. Summary of fitting time constants

3-2. Comparison with simulated flattening dynamics

Note S4: Radial distribution functions from the QM/MM MD simulations

Note S5: Energetics from solvent heating

Note S6: SVD analysis of isotropic and anisotropic scattering data

Note S7: Vibrational analysis of excited state simulations

Note S0: Data collection and reduction of time-resolved XSS and XES

In the pump-probe experiment at SACLA, the data were recorded by scanning an optical delay line with a motorized stage. At each delay stage position, a shutter of the optical laser was operated with 15 Hz, which was half of the XFEL repetition rate (30 Hz), to collect alternating laser-on and laser-off shots. For laser-on shots collected at time delays ≤ 2.5 ps, the time is corrected by post-sorting into 30 fs time bins using the timing diagnostics based on the X-ray beam branching scheme[1,2]. The jitter correction is not applied for laser-on shots at time delays longer than 2.5 ps to avoid degradation of the signal-to-noise ratio (S/N) by fine time binning. Therefore, only the data at > 2.5 ps retain a jitter of ~300 fs in root-mean-square. The shot-to-shot intensity ratio between the incident and fluorescent X-rays was monitored with photodiodes, removing outlier shots due to jet instability in the following XES and XSS analyses.

XSS: For each time delay, we collect single-shot outputs of a short-working-distance octal (SWD octal) multiport charge-coupled device (MPCCD) detector from 100 consecutive laser-on shots. After subtracting a dark background measured separately without XFEL pulses, these single-shot outputs are summed up to construct a laser-on image. A laser-off image is created in the same manner using laser-off shots adjacent to the corresponding laser-on shots. In the construction of the last laser-on and laser-off images, 100–200 shots are used depending on the number of excess shots. This procedure produces a data set consisting of ~45 pairs of laser-on and laser-off images at each time delay. We mask shadowed pixels and dead areas of these images. The sample–detector distance and detector position are calibrated using Debye Scherrer rings of a LaB_6 powder, which was loaded in a glass capillary (100 μm inner diameter) attached to an injection tip. After correcting for X-ray polarization and solid angle coverage per radial bin, the individual laser-on and laser-off images are scaled to the intensity integral of a corresponding simulated scattering curve over a range between 1.0 and 4.1 $Å^{-1}$ in the length of the scattering vector q, around two isosbestic points

(2.0 Å^{-1} and 3.1 Å^{-1}). The simulated curve is calculated as a sum of coherent and incoherent scattering arising from a liquid unit cell, *i.e.*, from one solute and 191 solvent molecules, corresponding to the smallest stoichiometry unit of a sample consisting of 100 mM $[Cu(dmphen)_2]PF_6$ in acetonitrile. The coherent scattering arising from the solute is calculated through the Debye equation using isolated $[Cu(dmphen)_2]^+$ and PF_6^- structures obtained from the literature[3,4], while the parameters by Hajdu[5] are used to calculate the incoherent scattering. For the solvent, reference data (up to ~8 Å^{-1}) of neat acetonitrile measured in a separate experiment are used to model the coherent and incoherent scattering. After the scaling, difference scattering images are acquired by subtracting laser-off images from laser-on images and are azimuthally integrated to extract one-dimensional isotropic and anisotropic difference scattering curves ($\Delta S(q,t)$ and $\Delta S_2(q,t)$). The anisotropic difference scattering signals are calculated with 12 azimuthal bins using the scheme described by Lorenz *et al.*[6] and Biasin *et al.*[7]. The difference scattering curves are averaged after removing outliers due to optical laser instability detected on the basis of the Chauvenet criterion[8–10].

XES: Laser-on and laser-off images are constructed from a single MPCCD detector with the same procedure employed in the XSS data collection, except that after the background subtraction, only pixel values over 70.0 analog-to-digital-units (ADU) in single-shot outputs are counted to reject the zero-photon peak, or the detector readout noise. This threshold corresponds to the detection of a single photon with an energy of 1.13 keV, well below actual photon energies detected in the experiment. From six regions of interest (ROIs) corresponding to the six crystal analyzers, a spectrum is constructed by summing up one-dimensional profiles after aligning the energy axis. The energy axis is calibrated using a static spectrum of $[Cu(dmphen)_2]^+$ measured at SPring-8 BL19XU. Each spectrum is normalized to the total integrated intensity. The laser-off spectra are averaged over the entire time range and used for the subtraction from laser-on spectra to yield difference spectra. The ~45 difference spectra at each time delay are averaged and then

used in the singular value decomposition (SVD) analysis.

Note S1: SVD analysis of time-resolved Cu Kα XES difference spectra

Figure S1 presents the results of the SVD analysis of the time-resolved Cu Kα XES difference spectra. In Figure S1b, the magnitudes of the singular values reveal that a single component dominates the transient signal. In Figure S1c, the right singular vectors (RSVs) indicate no trend with respect to time delay except for the first component. These observations demonstrate that only the first component is relevant for describing the changes in the Cu Kα XES difference spectrum.

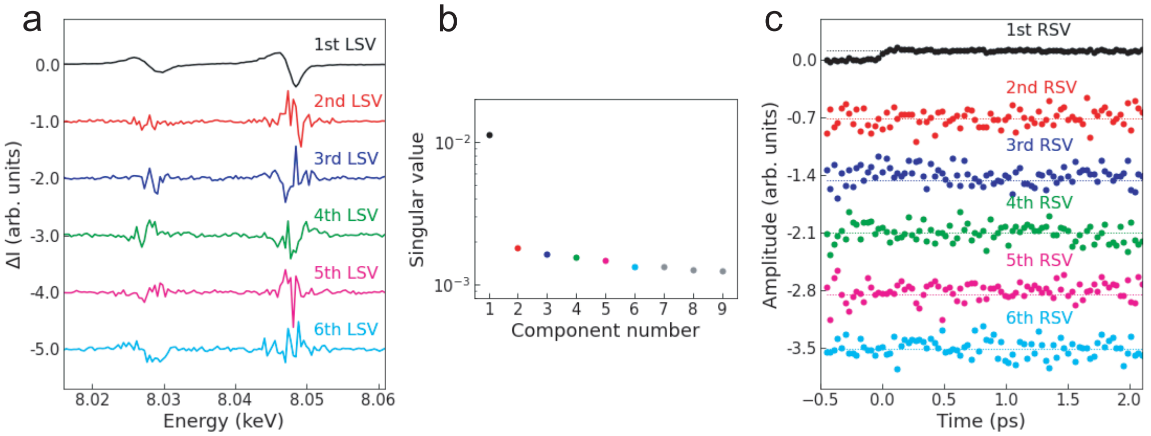

Figure S1. SVD components of the time-resolved XES data. (a) Left singular vectors (LSVs) of the 1st–6th components. (b) Singular value of each component. (c) Right singular vectors (RSVs) of the 1st–6th components. Dotted lines correspond to the magnitudes at 18.8 ps.

The 1st left singular vector (LSV) is associated with the change of Cu 3*d* electronic configuration (Cu^{1+}: d^{10} → Cu^{2+}: d^9), due to the metal-to-ligand-charge-transfer (MLCT) transition. The corresponding photoexcitation process promotes an electron from the ground state (S_0) into the lowest singlet (S_1) MLCT state and occurs within the instrumental response function (IRF). Therefore, the rise time of the 1st RSV reflects the overall time resolution of the pump-probe

experiment. Since the 1st RSV exhibits no clear decay within the measurement time window, we describe the temporal evolution of the 1st RSV ($I_{RSV}(t)$) as follows:

$$I_{RSV}(t) = A \cdot H(t - t_0) \otimes IRF(\sigma_{IRF}, t) \quad \text{(S1)}$$

Here A is the magnitude, t_0 is time zero, $H(t - t_0)$ is the Heaviside step function, $\otimes$ is the convolution operator, and $IRF(\sigma_{IRF}, t)$ is a Gaussian function with a width of σ_{IRF}. The least-squares fit is performed by a standard χ^2 minimization and uncertainties are at the 68% confidence level. The resultant fitted curve is shown in Figure 2 of the main text and the corresponding fitting parameters are summarized in Table S1. The width σ_{IRF} obtained here is used in the kinetic fitting of the time-dependent structural parameters extracted from the analysis of the time-resolved XSS data (Note S3).

Table S1. Fitting results of 1st RSV. The digits in parentheses are uncertainties.

σ_{IRF} / fs	t_0 / fs	A
28.7 (7.0)	−10.8 (5.0)	1.17×10^{-1} (1.25×10^{-3})

The lack of decrease in the total MLCT population within < 20 ps is consistent with the estimate based on the absolute radiative rate constants reported by Iwamura *et al*[11]. The highest radiative constant (k_r) reported in this study is 3.6×10^{7} s^{-1}, corresponding to the decay of the singlet MLCT (S_2) state. Assuming that all MLCT states decay with this radiative constant, the MLCT population decreases by < 0.1% at 20 ps. Although this estimate takes into account radiative de-excitation only, the nonradiative decay constants (k_{nr}) from MLCT states to the S_0 state are comparable to the highest radiative constant[12–14]; thus, the MLCT population will not decrease significantly through nonradiative de-excitation pathways within 20 ps. Consequently, this assessment corroborates the validity of our approximation that the MLCT population is constant within the time window of < 20 ps.

In order to determine the absolute MLCT population, the static Cu Kα XES spectra of the $[Cu(dmphen)_2]PF_6$ (Cu^{1+}: d^{10}) and $[Cu(dmphen)_2](PF_6)_2$ (Cu^{2+}: d^9) complexes in acetonitrile were measured in a separate experiment at the European XFEL (Figure S2). These Cu(I) and Cu(II) species should show Cu Kα emissions corresponding to 0% and 100% optical excitation in the pump-probe experiment at SACLA, respectively. After calibrating the energy axis, the Cu(I) and Cu(II) spectra measured at the European XFEL are interpolated and vertically offset, such that spacing between data points and the elastic scattering background underlying the emission signals are aligned to those in the pump-probe experiment at SACLA. This data correction is necessary to compare spectra measured with different instruments at the European XFEL and SACLA. Subsequently, the corrected Cu(I) and Cu(II) spectra are normalized to the total integrated intensity and their difference is calculated as the Cu(II) minus the Cu(I) signal. The obtained difference spectrum corresponds to the case where the solvated $[Cu(dmphen)_2]^+$ complex is 100% photoexcited in the pump-probe experiment; thus, it is scaled to the transient spectrum averaged over time intervals above 0.5 ps (Figure S2b), obtaining a fraction of molecules in MLCT states (MLCT population) in the pump-probe experiment of $0.202 \pm 2.40 \times 10^{-3}$.

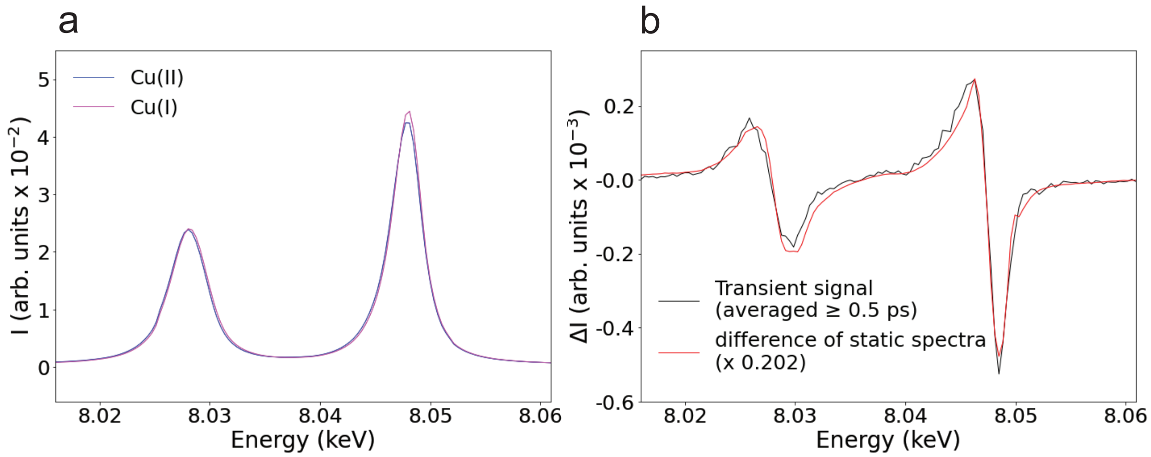

Figure S2. Determination of the absolute MLCT population. (a) Static Cu Kα XES spectra of the $[Cu(dmphen)_2]PF_6$ (Cu(I)) and $[Cu(dmphen)_2](PF_6)_2$ (Cu(II)) complexes after the data correction and normalization. (b) Red line is the scaled difference spectrum calculated using the static spectra in (a), while the black line is the transient spectrum averaged over time intervals above 0.5 ps.

Note S2: Structural analysis of time-resolved XSS difference signals

2-1. Simulations of $\Delta\boldsymbol{S}^{solute-solute}$, $\Delta\boldsymbol{S}^{solute-solvent}$, and $\Delta\boldsymbol{S}^{solvent-solvent}$

$\Delta\boldsymbol{S}^{solute-solute}$: The scattering signal arising from structural changes of the solute used in the fitting of the time-resolved XSS data is simulated as the difference between the scattering signals from putative ground and excited state structures. These are derived from the S_0 and the lowest triplet (T_1) MLCT state structures optimized with density functional theory (DFT) and time-dependent DFT (TD-DFT), respectively. The DFT and TD-DFT calculations are performed with the ORCA 5.0.1 program package using the PBE0 functional and the def2-SVP and def2-TZVP basis sets for C, N, H atoms and for the Cu atom, respectively. In the XSS fitting, the ground state is constrained to the DFT-optimized S_0 geometry, while the excited MLCT states have intermediate S_0–T_1 structures covering a wide range of the NNCuNN dihedral angles θ and Cu–N average distance r. Here, θ is defined as the angle between two planes, each of which consists of three atoms (two are the N atoms on one phenanthroline ligand and the last one is the central Cu atom). The excited state structures are generated according to the following procedure. First, 99 structures are generated through the image dependent pair potential (idpp) method[15] with a maximum force of 0.001 Å/fs on each atom using the S_0 and T_1 optimized geometries as initial and final structures. Then, the Cartesian coordinates of the nearest idpp structures are linearly interpolated. In this way, the atomic distance between atoms i and j of an intermediate structure is approximately a linearly interpolated distance between the S_0 and T_1 structures:

$$d_{ij}^{inter}(t) \approx d_{ij}^{S_0} + \Delta PJT(t)(d_{ij}^{T_1} - d_{ij}^{S_0}) \qquad \text{(S2)}$$

$\Delta PJT(t)$ in equation S2 is a scalar parameter that can take any value between 0 and 1, and can be

defined as a flattening coordinate representing the progress of the pseudo-Jahn–Teller (PJT) distortion. Finally, in order to account for the breathing motion dominating the coherent nuclear wave packet[16,17], for an intermediate structure with a given value of $\Delta PJT(t)$, the atoms of each phenanthroline ligand are displaced by a scalar parameter $\Delta R(t)$ along the vector from the central Cu atom to the center of mass of the ligand. The excited state structures generated according to this procedure span any dihedral angle θ between the S_0 and T_1 structures and a large range of the Cu–N average distance *r*.

The scattering signals from the S_0 and putative MLCT state structures used in the structural fitting are calculated through the Debye equation:

$$S_{sim}^{solute\text{-}solute}(q) = \sum_i^N f_i^2(q) + \sum_i^N \sum_{j\neq i}^N f_i(q) \cdot f_j(q) \cdot \frac{sin(q\cdot d_{ij})}{q\cdot d_{ij}} \qquad \text{(S3)}$$

N is the number of atoms of $[Cu(dmphen)_2]^+$, $f_i(q)$ is the atomic form factor of atom i, and d_{ij} are the atom pair distances of the solute in the ground or an intermediate excited state geometry (see eq. S2). After the fitting, at each time delay, the change in the average Cu–N bond length $\Delta r(t)$ and the change in the dihedral angle between the two phenanthroline ligands $\Delta\theta(t)$ are extracted from the S_0 and the intermediate MLCT structures and used in Figure 4 of the main text.

$\boldsymbol{\Delta S^{solvent-solvent}}$: The bulk solvent signal arises from the changes in solvent–solvent atomic pair distances and is typically approximated as follows:

$$\Delta S^{solvent-solvent}(q,t) = \Delta T(t) \cdot \frac{\partial\left(\Delta S_{ref}^{solvent}(q)\right)}{\partial T} + \Delta\rho(t) \cdot \frac{\partial\left(\Delta S_{ref}^{solvent}(q)\right)}{\partial \rho} \qquad \text{(S4)}$$

Here, $\frac{\partial\left(\Delta S_{ref}^{solvent}(q)\right)}{\partial T}$ and $\frac{\partial\left(\Delta S_{ref}^{solvent}(q)\right)}{\partial \rho}$ are the scattering signal differentials arising from a temperature change at a constant density and from a density change at a constant temperature, respectively. Since thermal expansion is expected to occur on a much longer timescale (>> 100

ps)[18] than the time window of the present pump-probe experiment, $\Delta S^{solvent-solvent}$ can be reduced to the impulsive heating as follows:

$$\Delta S^{solvent-solvent}(q,t) \cong \Delta T(t) \cdot \frac{\partial\left(\Delta S_{ref}^{solvent}(q)\right)}{\partial T} \qquad \text{(S5)}$$

We measure $\frac{\partial\left(\Delta S_{ref}^{solvent}(q)\right)}{\partial T}$ separately using a 5 mM acetonitrile solution of a dye (4-(N,N-diethylamino)-2-methoxy-4’-nitro-azobenzene; CAS 6373-95-1) at 100 ps after photoexcitation by 550 nm optical pulses, when most dye molecules decay into the ground state and the structural signal relevant to the solute can be neglected as described elsewhere[19]. The impulsive heating signal obtained in the reference measurements (Figure S3) agrees well with the literature[19] and is employed in the structural fitting analysis.

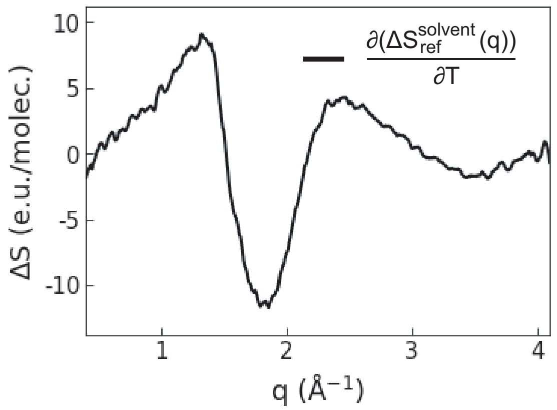

Figure S3. Impulsive heating signal $\frac{\partial\left(\Delta S_{ref}^{solvent}(q)\right)}{\partial T}$ of bulk liquid acetonitrile at ΔT = 1 K.

$\Delta S^{solute-solvent}$: The difference scattering signal arising from changes in the solute–solvent atom pair distances is extracted from quantum mechanics/molecular mechanics (QM/MM) molecular dynamics (MD) simulations of $[Cu(dmphen)_2]^+$ after photoexcitation to the S_1 MLCT state in acetonitrile (details of the simulations reported elsewhere[3]). Figure S4a shows a difference scattering signal $\Delta S_{BOMD}^{solute\text{-}solvent}$ calculated using the time-dependent solute–solvent radial

distribution functions (RDFs) averaged over the MLCT QM/MM MD trajectories at each time step and the RDFs of equilibrium ground state trajectories. $\Delta S_{BOMD}^{solute\text{-}solvent}$ shows no significant time dependence after 3 ps, indicating that the solute–solvent RDFs in the MLCT QM/MM MD trajectories are equilibrated after this time.

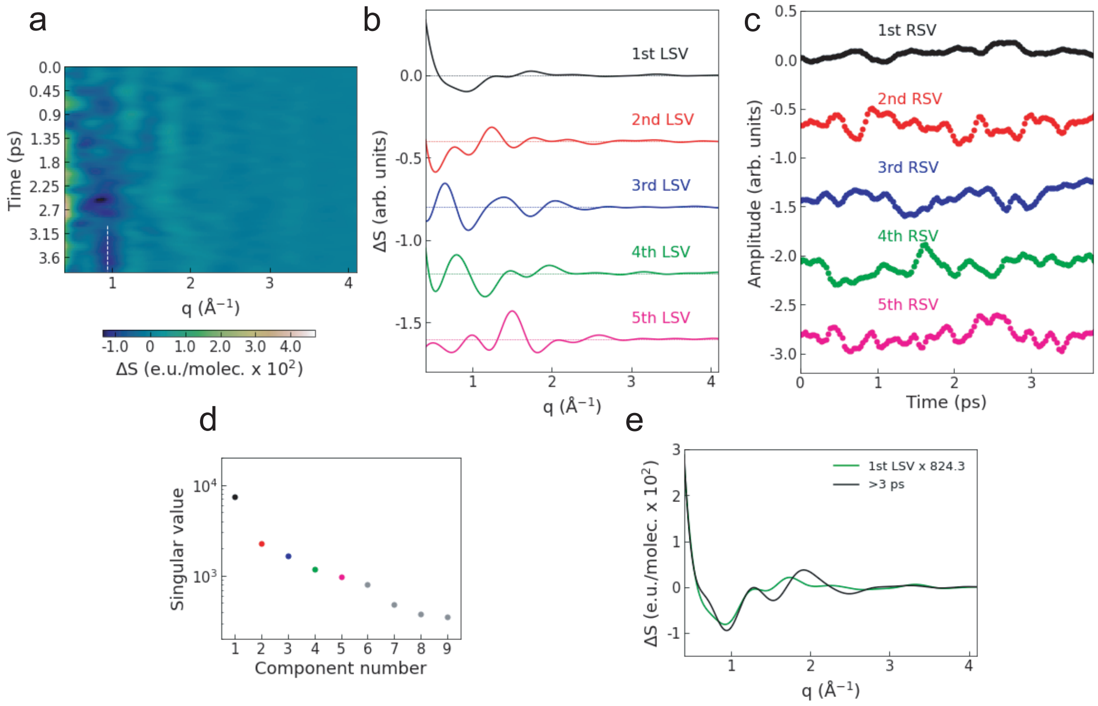

Figure S4. $\Delta S_{BOMD}^{solute\text{-}solvent}$ signal. (a) Difference scattering signal $\Delta S_{BOMD}^{solute\text{-}solvent}$ calculated using time-dependent solute–solvent radial distribution functions (RDFs) from the QM/MM MD simulations. The white dashed line at 0.94 Å^{-1} indicates the position of the negative peak, which stabilizes after equilibration of the QM/MM MD trajectories. (b) 1st–5th LSVs of the SVD of $\Delta S_{BOMD}^{solute\text{-}solvent}$. (c) 1st–5th RSVs of the SVD. (d) Singular value of each SVD component. (e) $\Delta S_{sim}^{solute\text{-}solvent}$ (black) calculated using the RDFs of equilibrated ground and excited state QM/MM MD trajectories and the scaled 1st LSV (green) of the SVD of $\Delta S_{BOMD}^{solute\text{-}solvent}$.

We employ the scattering signal calculated from the RDFs of the ground state equilibrated trajectories and the excited state trajectories considering only time delays over 3 ps as a fitting component $\Delta S_{sim}^{solute\text{-}solvent}$. To assess the approach of using $\Delta S_{sim}^{solute\text{-}solvent}$ as a single solute–solvent component at all time delays in the structural fitting, we performed an SVD analysis of $\Delta S_{BOMD}^{solute\text{-}solvent}$ (Figure S4b–d). First of all, the 1st LSV has a similar shape as $\Delta S_{sim}^{solute\text{-}solvent}$

(Figure S4e), therefore, $\Delta S_{sim}^{solute\text{-}solvent}$ can account for the changes in the signal associated with this SVD component. Furthermore, only incorporation of $\Delta S_{sim}^{solute\text{-}solvent}$ in the model leads to a significant improvement of the structural fitting, while any extra solute–solvent signal (*i.e.*, one of the 2nd–5th LSVs shown in Figure S4b) is found to negligibly improve the quality of the fitting (see next section and Figure S6). These observations corroborate the validity of choosing $\Delta S_{sim}^{solute\text{-}solvent}$ as the only solute–solvent fitting component.

2-2. Sensitivity of XSS to structural degrees of freedom

The scattering signals arising from different structural degrees of freedom should be sufficiently different to enable a robust structural determination. To confirm this prerequisite, we have calculated the difference scattering signals from a set of structures obtained by displacing the ligands of the S_0 DFT-optimized geometry to mimic the flattening ($\Delta\theta$) or breathing (ΔR) motions while keeping the other degrees of freedom fixed (Figure S5). The difference scattering patterns associated with ΔR and $\Delta\theta$ exhibit clear differences, and are both different from the scattering signal associated with the solvent response, corroborating the robustness of the structural fitting illustrated in the following section.

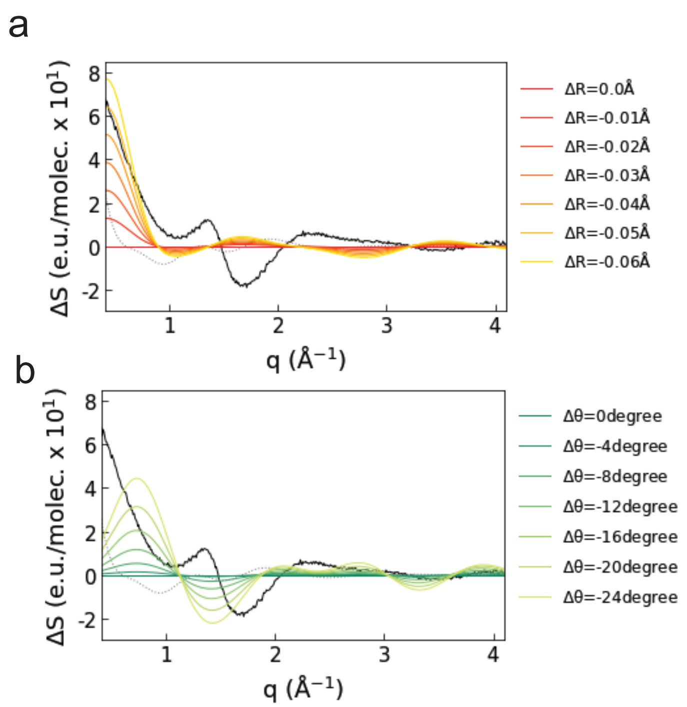

Figure S5. XSS sensitivity to ligand flattening, breathing and changes in the solvation structure. (a) The colored curves are the difference scattering signals calculated as the atoms of each phenanthroline ligand are moved by $\Delta R(t)$ along the vector from the central Cu atom to the center of mass of the ligand with the dihedral angle fixed to the value of the S_0 optimized geometry. (b) The colored curves are the difference scattering signals calculated as the dihedral angle is decreased by $\Delta\theta$ with the distance between Cu and ligand atoms fixed to the values of the S_0 optimized geometry. (a–b) The calculated signals are multiplied by the MLCT excited population, 0.202. The overlaid black solid and dotted curves correspond to the measured difference scattering signal at 18.8 ps and the fitted $\Delta S_{sim}^{solute-solvent}$ component, respectively.

2-3. Structural fitting

The structural fitting is performed by minimizing for each time delay the discrepancy between $\Delta S_{model}(q,t)$, consisting of the sum of the three components described above (see also equation 2 in the main text), and the experimental data $\Delta S(q,t)$ using a maximum likelihood estimation with the χ^2 estimator:

$$\chi^2 = \frac{1}{N_q - p - 1} \sum_q^{N_q} \frac{(\Delta S_{model}(q,t) - \Delta S(q,t))^2}{\sigma^2} \qquad \text{(S6)}$$

Here, N_q is the number of data points at each time delay t, p is the number of free parameters, and

σ is the standard deviation of the data. Prior to performing the structural fitting at each time delay, we investigate the contribution of the three components to the scattering signal at selected time delays as described in the following steps (i) and (ii).

(i) Identifying the presence of the solute–solvent signal $\Delta S_{sim}^{solute\text{-}solvent}$

In order to corroborate the importance of the solute–solvent contribution, we fit the difference scattering signal measured at the longest time delay (18.8 ps), when the structural evolution of the solute is completed, to two different models. One is composed of solute–solute, solute–solvent, and solvent–solvent terms (model_1), while the other excludes the solute–solvent term (model_2). Figure S6 clearly shows that the fitting quality is significantly improved when the model includes the solute–solvent term. This is further corroborated by the Akaike's Information Criterion (AIC)[20]. The AIC score is given as follows:

$$AIC = -2 \cdot \ln(L) + 2p \qquad \text{(S7)}$$

Here, L is the likelihood of the fitting with a parameter set. Using the converged parameter sets obtained by fitting the data with model_1 and model_2, we calculate the AIC difference ($\Delta AIC = AIC_{model\_2} - AIC_{model\_1}$) as 2.9×10^4. Since the model with the lower AIC score offers the better fit, the positive AIC difference indicates that model_1 is superior to model_2, demonstrating the necessity of the solute–solvent term to adequately reproduce the measured data.

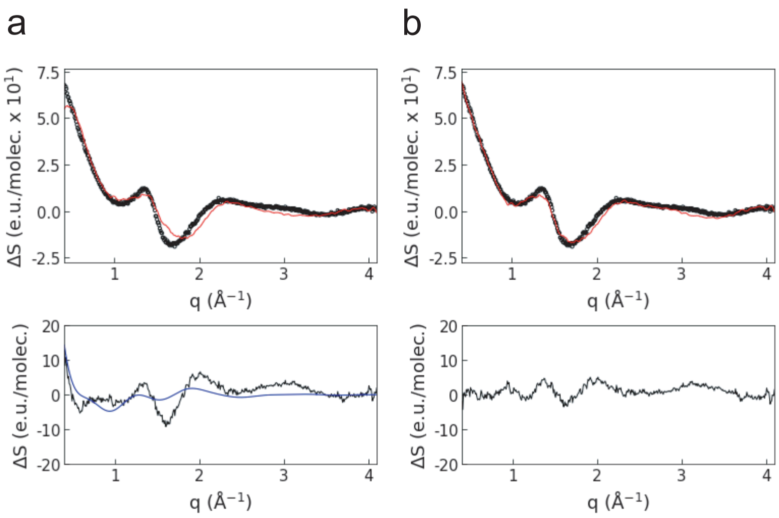

Figure S6. Fitting of the difference scattering signal measured at 18.8 ps. (a) Fitting model includes solute–solute and solvent–solvent terms (model_2). (b) Fitting model includes solute–solute, solute–solvent, and solvent–solvent terms (model_1). (top, a–b) Black circles and the red line are the difference scattering signal and the fitted curves, respectively. (bottom, a–b) Residual of the fitting and the scaled simulated solute–solvent signal ($\Delta S_{sim}^{solute\text{-}solvent} \times 0.05$) are shown as black and blue lines, respectively.

(ii) Contributions from 2nd–6th LSVs of the SVD of $\Delta S_{BOMD}^{solute\text{-}solvent}$

The data measured at several time delays are fitted using a model assuming two different solute–solvent terms (model_3), as described below:

$$\Delta S_{model\_3}(q) = A_{MLCT} \cdot \{\Delta S_{sim}^{solute\text{-}solute}(\Delta\theta, \Delta r) + \alpha \cdot \Delta S_{sim}^{solute\text{-}solvent}(q) + \beta \cdot \Delta S_{sim}^{extra-solute-solvent}(q)\} + \Delta T \cdot \frac{\partial\left(\Delta S_{ref}^{solvent}(q)\right)}{\partial T} \quad \text{(S8)}$$

Here, $\Delta S_{sim}^{solute\text{-}solvent}$ corresponds to the black line in Figure S4e, *i.e.* the solute–solvent scattering signal calculated using equilibrium solute–solvent RDFs from the QM/MM MD simulations, while $\Delta S_{sim}^{extra-solute-solvent}$ is one of the 2nd–5th LSVs of the SVD of $\Delta S_{BOMD}^{solute\text{-}solvent}$, obtained from the time-dependent RDFs. α and β are scaling factors, which are positively constrained. The fitting results (Figure S7a–d) clearly show that the magnitudes of the fitted 2nd–5th LSVs are negligibly small at 0.3 ps. Figure S7e shows the AIC difference ($\Delta AIC = AIC_{model\_3} - AIC_{model\_1}$) as a

function of time delays. Since the AIC differences are mostly positive through 2nd–5th LSVs, the extra solute–solvent component in model_3 is redundant, supporting the conclusion that the solute–solvent contribution is well characterized by the sole $\Delta S_{sim}^{solute\text{-}solvent}$ component.

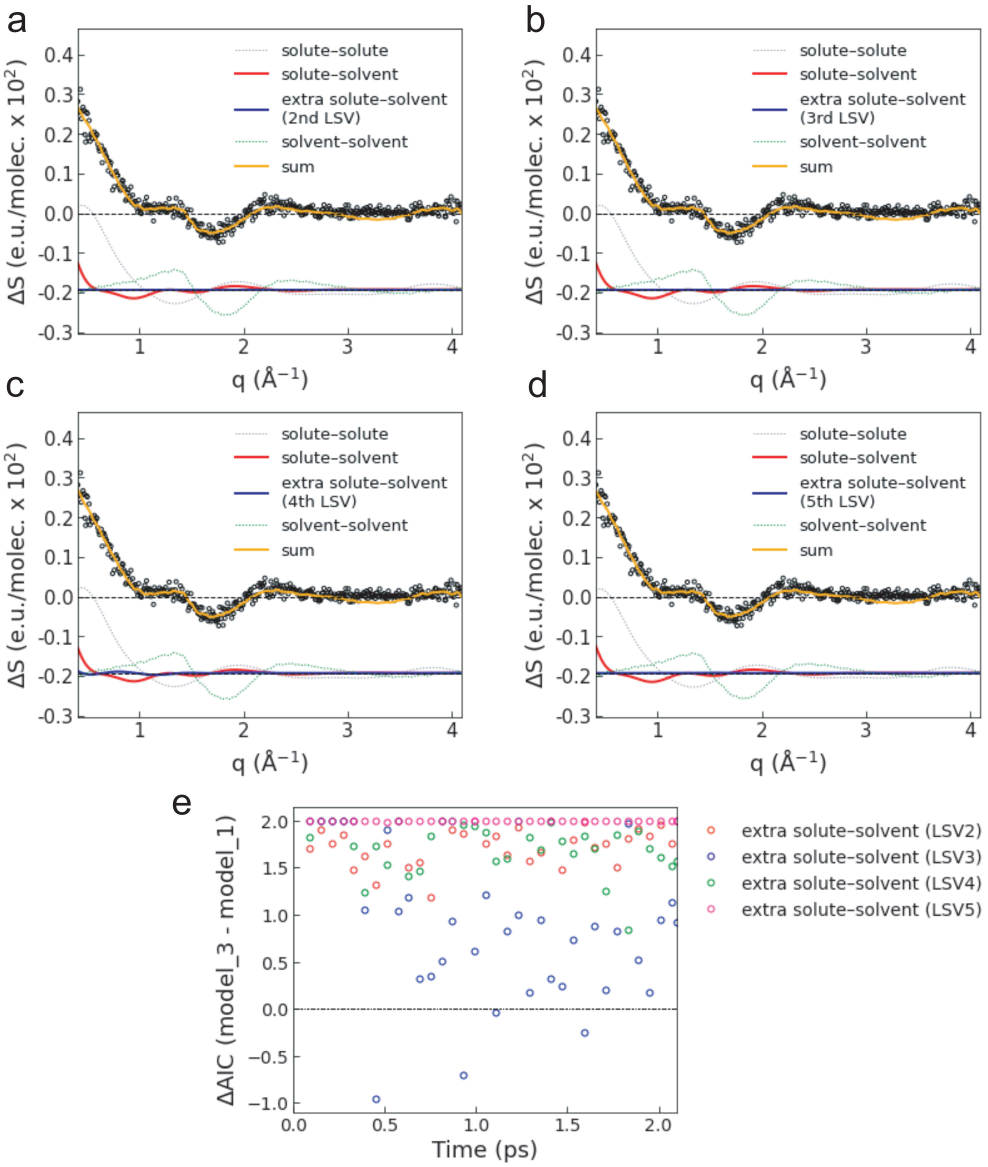

Figure S7. Contributions from 2nd–5th LSVs of the SVD of $\Delta S_{BOMD}^{solute\text{-}solvent}$. (a–d) Fitting of the difference scattering signal at 0.3 ps with model_3. The magnitudes of $\Delta S_{sim}^{extra-solute-solvent}$ after the fit (blue solid lines) are smaller than $\Delta S_{sim}^{solute\text{-}solvent}$ (red solid lines). (e) Colored circles are the AIC differences ($\Delta AIC = AIC_{model\_3} - AIC_{model\_1}$) as a function of time delays. Except a few points, most of the ΔAIC values are positive.

Steps (i) and (ii) are finally followed by the structural fitting at each time delay (model_1). The resulting fitted curves are shown in Figure 3 of the main text. Figure S8 shows the contributions of the three components of the model for the fitting at selected time delays and the contours of the likelihood distribution function enclosing 68% of the probability distribution, which show the correlation between the different parameters. The correlation between $\Delta PJT(t)$ and $\Delta R(t)$ is strong because both parameters change the Cu–N bond lengths.

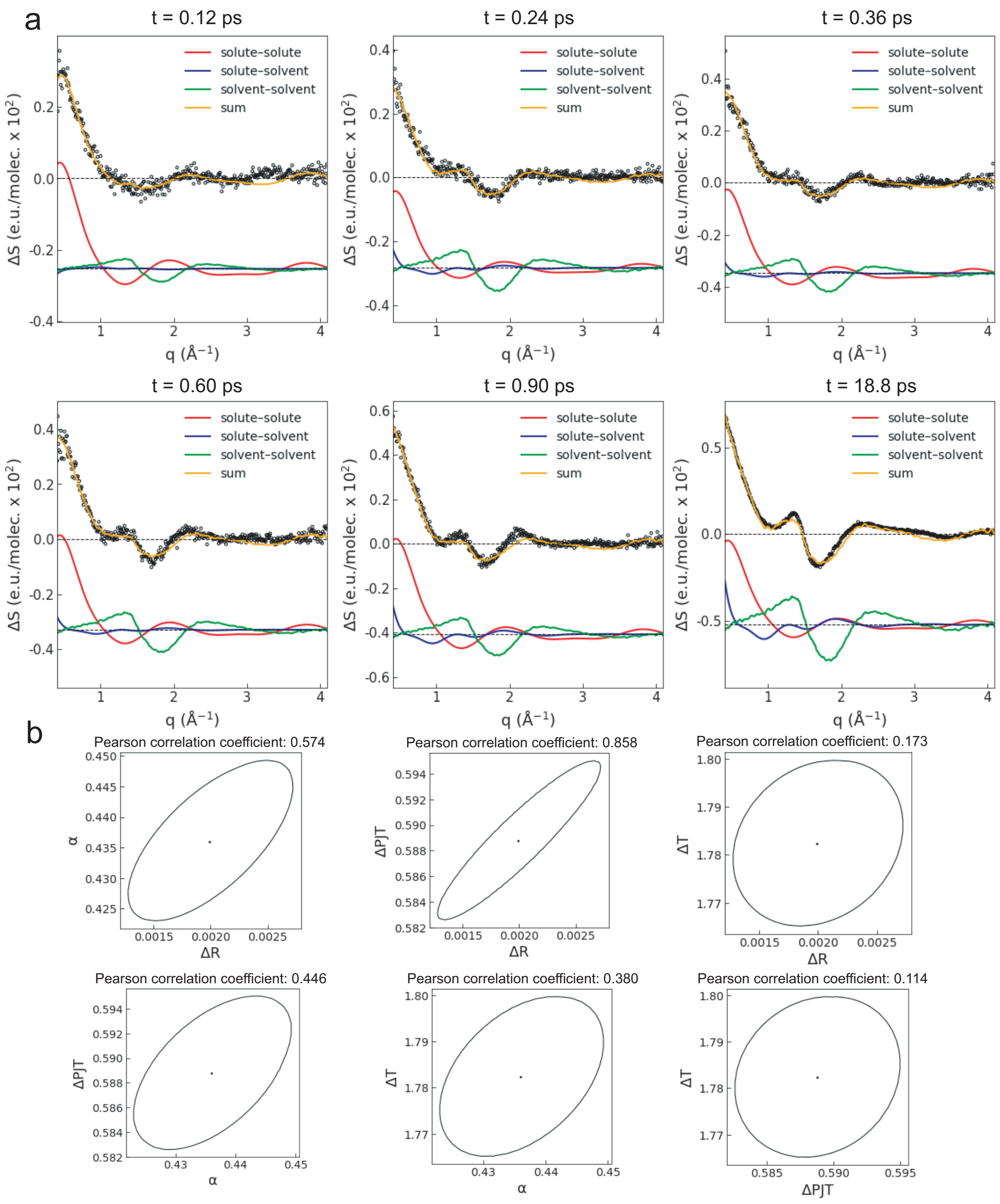

Figure S8. Results of the structural fitting. (a) Contributions to the fitting of the three components of the model at selected time delays. (b) Contours of the likelihood distribution function enclosing 68% of the probability distribution, showing the correlation between the parameters at 18.8 ps.

To test the use of a solute–solvent component calculated from the equilibrated parts of the excited state QM/MM MD trajectories ($\Delta S_{sim}^{solute\text{-}solvent}$) for fitting the data at short time delays,

when the system is in nonequilibrium states, we conducted another fitting using the 1st LSV of the SVD analysis of $\Delta S_{BOMD}^{solute\text{-}solvent}$ instead of $\Delta S_{sim}^{solute\text{-}solvent}$ as the solute–solvent component. The results of the fit are shown in Figure S9a.

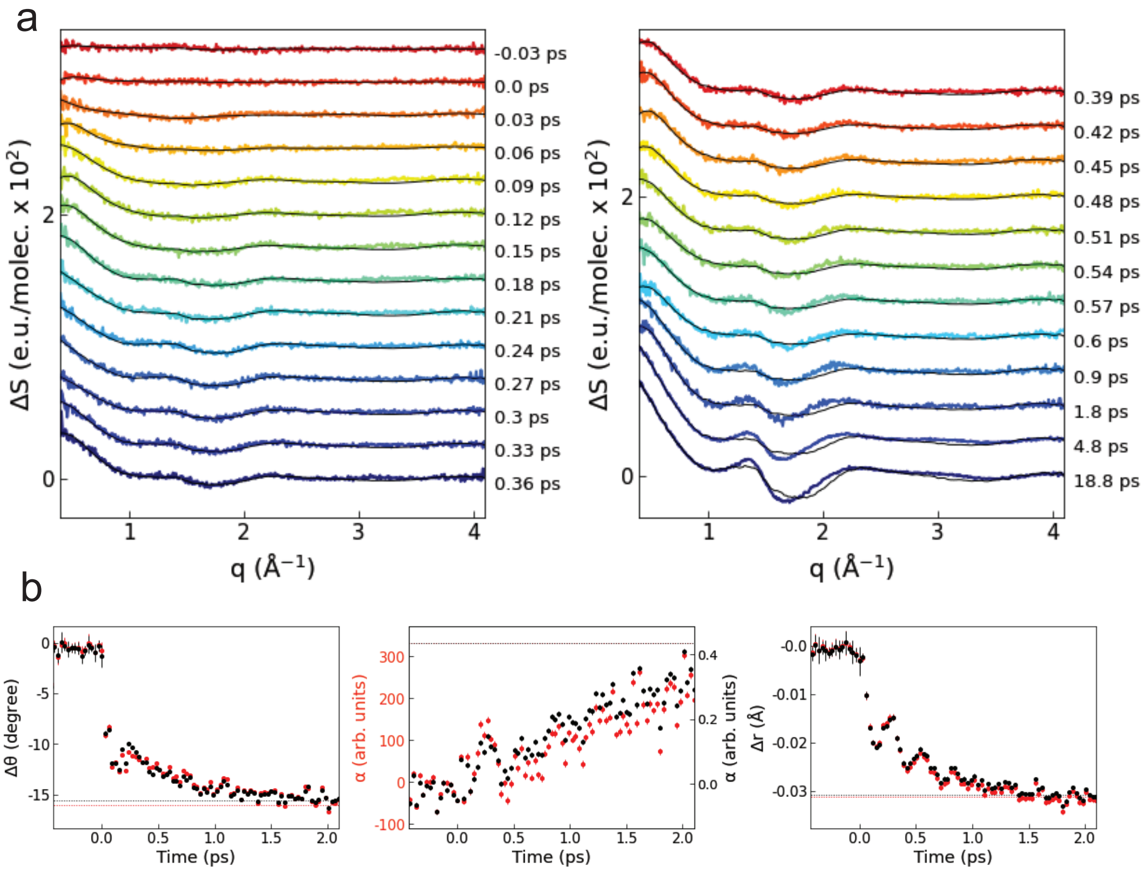

Figure S9. Fitting using the 1st LSV of the SVD analysis of $\Delta S_{BOMD}^{solute\text{-}solvent}$ as the solute–solvent component. (a) Colored lines are the experimental data (vertically offset for clarity), while the overlaid black lines correspond to the fitted curves. (b) Optimized structural parameters as a function of time delays. The black and red circles are extracted from the fittings using $\Delta S_{sim}^{solute\text{-}solvent}$ and the 1st LSV of the SVD analysis of $\Delta S_{BOMD}^{solute\text{-}solvent}$ as the solute–solvent component, respectively. Dotted lines correspond to the values at 18.8 ps.

Very little difference is observed in the evolution of the optimized structural parameters (Figure S9b), indicating that the structural fitting is largely insensitive to the small difference (especially at q > 1.5 Å^{-1}) between the 1st LSV of the SVD analysis of $\Delta S_{BOMD}^{solute\text{-}solvent}$ and $\Delta S_{sim}^{solute\text{-}solvent}$ (Figure S4e). Moreover, the fitting quality using $\Delta S_{sim}^{solute\text{-}solvent}$ (Figure 3 in the main text) is

better at long time delays than when using the 1st LSV of the SVD analysis of $\Delta S_{BOMD}^{solute-solvent}$ (Figure S9a), which shows that $\Delta S_{sim}^{solute-solvent}$ can explain the experimental data more adequately.

2-4. Fitting summary

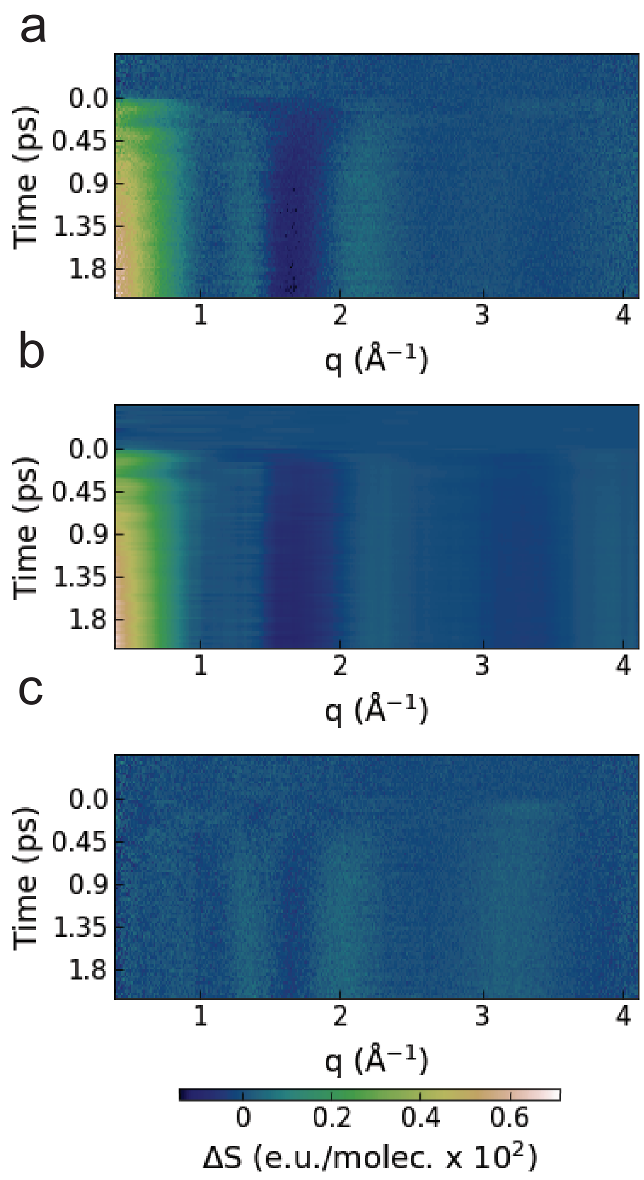

Figure S10. Two-dimensional scattering signals. (a) Measured data. (b) Fitted curves. (c) Residuals.

Table S2 presents the Cartesian coordinates of the equilibrated MLCT state, corresponding to the excited state structure that best fits the data at 18.8 ps.

Table S2. Cartesian coordinate of the MLCT state at 18.8 ps.

Element	X / Å	Y / Å	Z / Å
Cu	-2.71969	0.60191	-0.39621
N	-1.52237	1.12511	-0.75207
N	-1.52248	-1.09419	0.79401
C	-3.88075	2.28254	-1.66453
C	-3.88097	-2.24983	1.70852
C	-2.71969	0.60191	-0.39621
C	-2.71972	-0.56997	0.43936
C	-1.46266	2.18208	-1.55647
C	-1.46283	-2.15118	1.59839
C	-3.94808	1.15741	-0.81310
C	-3.94817	-1.12435	0.85755
C	-2.65275	2.79580	-2.01530
C	-2.65302	-2.76389	2.05830
C	-5.17871	0.55801	-0.39234
C	-5.17874	-0.52450	0.43724
H	-2.58501	-3.64588	2.69808
H	-4.80085	-2.65783	2.13610
H	-6.11690	-0.94569	0.80657
H	-6.11683	0.97924	-0.76169
H	-4.80076	2.69186	-2.09057
H	-2.58448	3.67776	-2.65508
C	-0.12855	2.71803	-1.95278
H	0.25697	3.40571	-1.18385
H	0.60769	1.91331	-2.08026
H	-0.18244	3.27772	-2.89656
C	-0.12887	-2.68808	1.99370
H	0.25696	-3.37396	1.22338
H	0.60743	-1.88378	2.12347
H	-0.18249	-3.24990	2.93623
N	1.53851	1.04746	0.86996
N	1.53645	-1.01871	-0.83140
C	3.90476	2.14111	1.87485
C	3.90038	-2.11538	-1.83844
C	2.75198	0.56003	0.47165
C	2.75089	-0.53256	-0.43452
C	1.48245	2.04664	1.76468

C	1.47830	-2.01782	-1.72606
C	3.97823	1.09997	0.93990
C	3.97603	-1.07380	-0.90415
C	2.65595	2.61661	2.27478
C	2.65059	-2.58903	-2.23754
C	5.20633	0.52218	0.46969
C	5.20528	-0.49721	-0.43546
H	6.14686	0.90757	0.87162
H	6.14494	-0.88343	-0.83862
H	4.81404	-2.49422	-2.30311
H	2.56948	-3.38076	-2.98426
H	2.57654	3.40831	3.02171
H	4.81928	2.51813	2.33930
C	0.12622	-2.48494	-2.16079
H	-0.40041	-2.98703	-1.33426
H	-0.49587	-1.62970	-2.46811
H	0.19849	-3.19026	-2.99828
C	0.13138	2.51531	2.20087
H	-0.39544	3.01816	1.37492
H	-0.49145	1.66083	2.50882
H	0.20530	3.22046	3.03837

Note S3: Kinetic time constants from the XSS analysis

3-1. Summary of fitting time constants

The time evolutions of $\Delta r(t)$, $\Delta\theta(t)$, $\alpha(t)$, and $\Delta T(t)$ extracted from the structural analysis are fitted with phenomenological kinetic models by the least-squares method using the standard χ^2 estimator. The uncertainties are at 68% confidence level. The applied models are described by different combinations of a step function, a damped sine function, and exponential functions, which are convoluted by the Gaussian IRF obtained in Note S1. The fitting equations are the following:

$$\Delta r(t) = \left\{A_{step} + A_{exp1} \cdot (1 - e^{-\frac{t-t_0}{\tau_{exp1}}}) + A_{osc} \cdot e^{-\frac{t-t_0}{\tau_D}} \cdot \sin(\frac{2\pi(t-t_0)}{\tau_{osc}})\right\} \cdot H(t-t_0) \otimes IRF(\sigma_{IRF}, t)$$

$$\Delta \theta(t) = \left\{A_{step} + A_{exp1} \cdot (1 - e^{-\frac{t-t_0}{\tau_{exp1}}}) + A_{osc} \cdot e^{-\frac{t-t_0}{\tau_D}} \cdot \sin(\frac{2\pi(t-t_0)}{\tau_{osc}})\right\} \cdot H(t-t_0) \otimes IRF(\sigma_{IRF}, t)$$

$$\alpha(t) = \left\{A_{exp1} \cdot (1 - e^{-\frac{t-t_0}{\tau_{exp1}}}) + A_{osc} \cdot e^{-\frac{t-t_0}{\tau_D}} \cdot \sin(\frac{2\pi(t-t_0)}{\tau_{osc}})\right\} \cdot H(t-t_0) \otimes IRF(\sigma_{IRF}, t)$$

$$\Delta T(t) = \left\{A_{exp1} \cdot (1 - e^{-\frac{t-t_0}{\tau_{exp1}}}) + A_{exp2} \cdot (1 - e^{-\frac{t-t_0}{\tau_{exp2}}})\right\} \cdot H(t-t_0) \otimes IRF(\sigma_{IRF}, t) \quad \text{(S9)}$$

The kinetic time constants obtained from the fitting are summarized in Table S3.

Table S3. Kinetic time constants. The digits in parentheses are uncertainties.

Parameter	$\Delta r(t)$ / Å	$\Delta\theta(t)$ / Degree	$\alpha(t)$	$\Delta T(t)$ / K
A_{step}	-1.36×10^{-2} (6.84×10^{-4})	−8.82 (0.573)	-	-
A_{exp1}	-1.80×10^{-2} (5.95×10^{-4})	−6.78 (0.502)	0.425 (2.91×10^{-2})	1.22 (4.26×10^{-2})
τ_{exp1} / fs	582 (40.7)	554 (66.8)	1.40×10^{3} (169)	6.11×10^{3} (505)
A_{osci}	-8.96×10^{-3} (1.27×10^{-3})	−3.53 (1.42)	0.199 (6.23×10^{-2})	-
τ_{osc} / fs	286 (4.75)	401 (28.9)	319 (30.0)	-
τ_D / fs	350 (57.4)	203 (80.7)	220 (94.4)	-
A_{exp2}	-	-	-	0.601 (1.51×10^{-2})
τ_{exp2} / fs	-	-	-	125 (15.6)
t_0 / fs	60.3 (3.49)	10.5 (6.42)	185 (16.9)	14.4 (9.25)
σ_{IRF} / fs	28.7 (fixed)	28.7 (fixed)	28.7 (fixed)	28.7 (fixed)

For $\Delta r(t)$, the measured period of coherent oscillations (286 fs) agrees with the main period of oscillations observed in the evolution of the mean Cu–N distance obtained from the

excited state QM/MM MD simulations (~282 fs, see Figure S22 further below). This period is further close to the period of a breathing normal mode at the T_1 (301 fs) and S_1 (290 fs) optimized structures identified in vibrational analyses carried out using TD-DFT/CPCM calculations with PBE0 and variational excited state calculations with BLYP in vacuum, respectively (see also Figure S21 below). For $\Delta\theta(t)$, the measured period (401 fs) is significantly shorter than the period of a flattening mode (see Figure S21) obtained in the vibrational analyses (925 fs from TD-DFT/CPCM calculations at the T_1 optimized geometry and 926 fs from variational density functional calculations in vacuum). This discrepancy indicates that an accurate description of the motion along the flattening coordinate needs to consider the effect of surrounding solvent beyond the conductor-like polarizable continuum model.

$\Delta\theta(t)$ and $\alpha(t)$ obtained from the structural fitting are plotted together and overlaid in Figure S11. Clearly, the phase of the oscillations in $\alpha(t)$ has a delay compared to those in $\Delta\theta(t)$, indicating that the fast initial flattening triggers a delayed coherent solvent response.

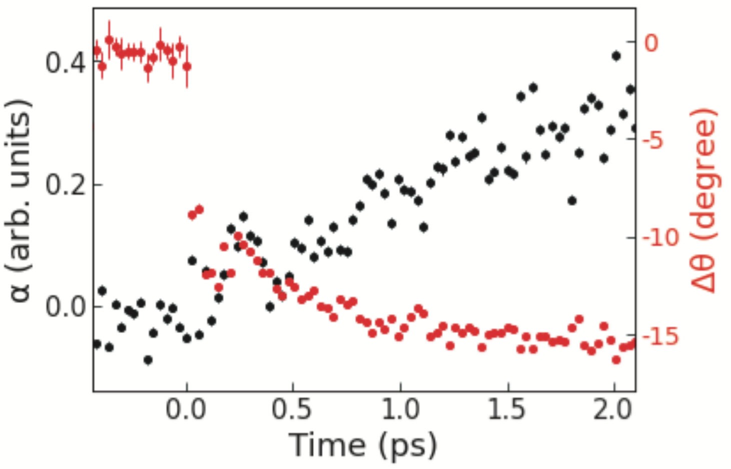

Figure S11. Overlaid plots of $\Delta\theta(t)$ and $\alpha(t)$ as obtained from the structural fitting.

3-2. Comparison with simulated flattening dynamics

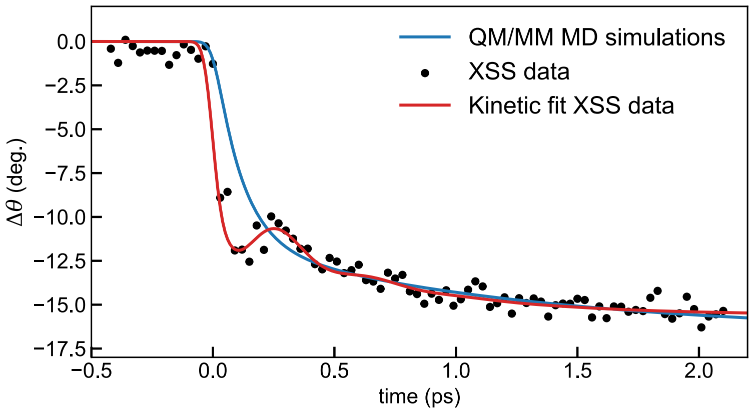

Figure S12. Comparison between the change in the NNCuNN dihedral angle Δ*θ*(t) from the experiment and from the simulations. (Black dots) Results of the structural analysis of the XSS data together with the kinetic fitting curve (red line). (Blue line) Biexponential fit of Δ*θ*(t) obtained as an average over nonequilibrium excited state QM/MM MD trajectories convoluted with the experimental IRF. The instantaneous average *θ*(t) is reported together with the parameters of the biexponential fit in ref. S3.

Note S4: Radial distribution functions from the QM/MM MD simulations

Figure S13 shows the equilibrium solute–solvent RDFs extracted from the QM/MM MD simulations[3] and used in the calculation of $\Delta S_{sim}^{solute\text{-}solvent}$. The small peak at ~5 (~5.5) Å in the ground state Cu–N (Cu–C) RDFs indicates a low degree of ordering in the first solvation shell. In the MLCT state, this peak disappears while the magnitude at around < 4 Å increases. The changes in RDFs reflect a higher probability to find acetonitrile molecules at around < 4 Å oriented preferentially with the N atom towards the Cu atom after photoexcitation, which is consistent with the nature of the MLCT states. Indeed, the flattening distortion creates an open space that enables acetonitrile molecules to come closer to the Cu atom, which is confirmed by the time-dependent differences in the RDFs as shown in Figure S14. Accordingly, the scaling factor $\alpha(t)$ is diagnostic of how close acetonitrile molecules get to the Cu atom.

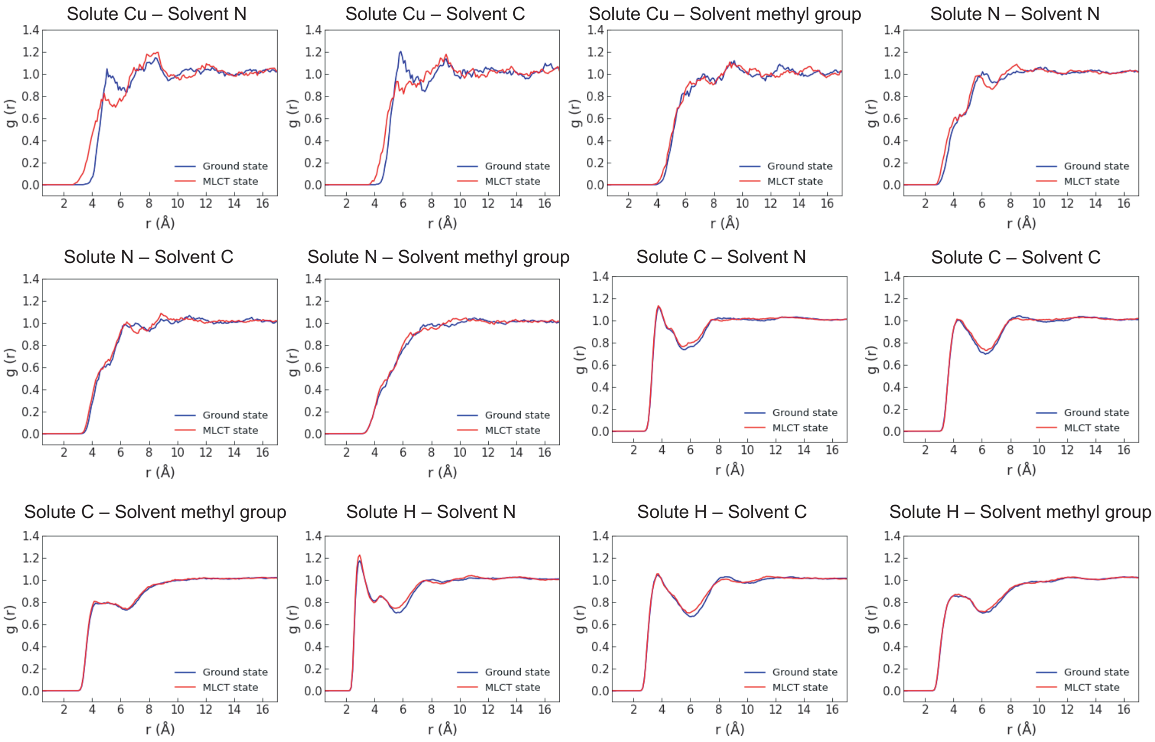

Figure S13. Solute–solvent radial distribution functions from the QM/MM MD simulations.

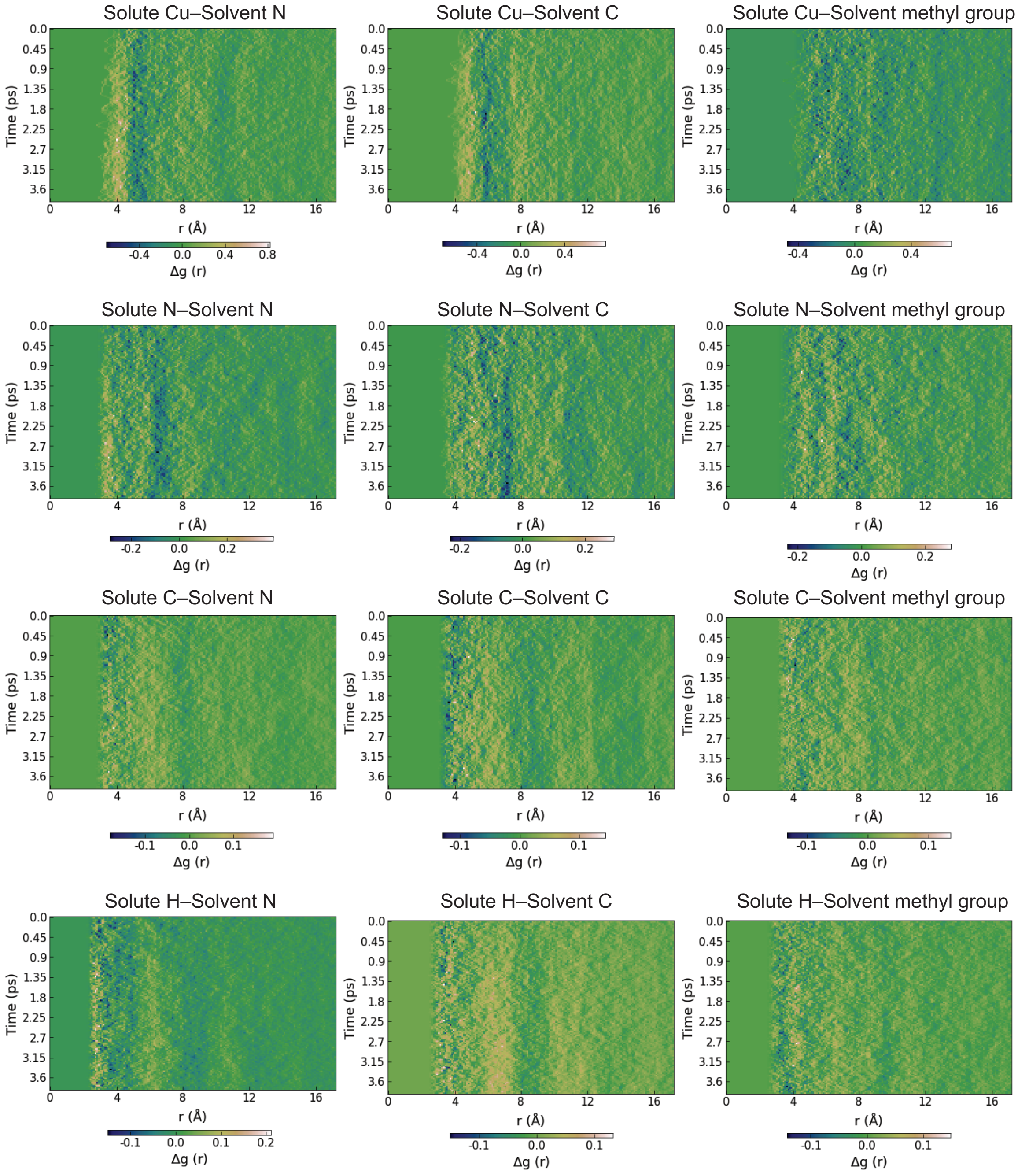

Figure S14. Time-dependent differences in radial distribution functions from the QM/MM MD simulations.

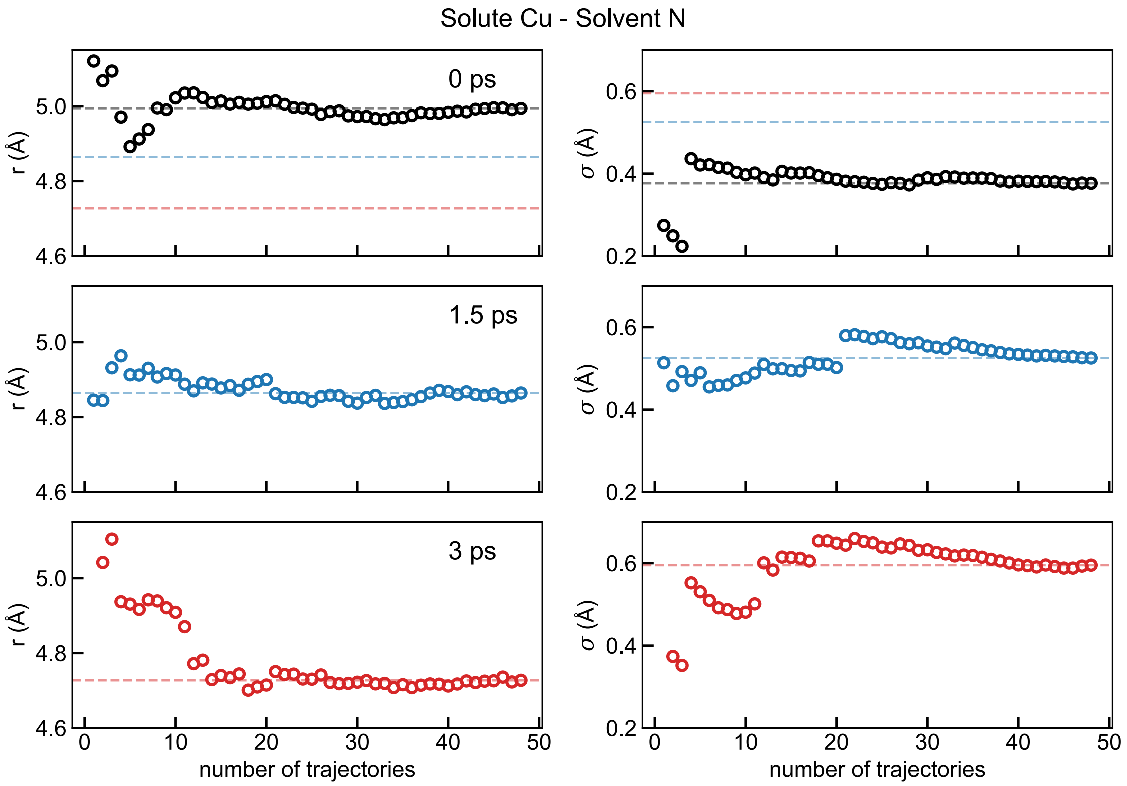

Figure S15. Convergence of simulated excited state solute–solvent radial distribution functions with respect to number of excited state QM/MM MD trajectories. (Left) Mean distance between the Cu atom and the N atom of acetonitrile molecules calculated from the Cu–N RDF for r(Cu–N) < 5.65 Å (includes the first peak in the RDF) at different times as a function of the number of excited state trajectories used to obtain the RDF. (Right) Standard deviation of the Cu–N distance calculated using the Cu–N RDF for r(Cu–N) < 5.65 Å as a function of the number of excited state trajectories.

Note S5: Energetics from solvent heating

The time evolution of $\Delta T(t)$ obtained from the fitting of the time-resolved XSS data is not discussed in the main text but is presented in Figure S16.

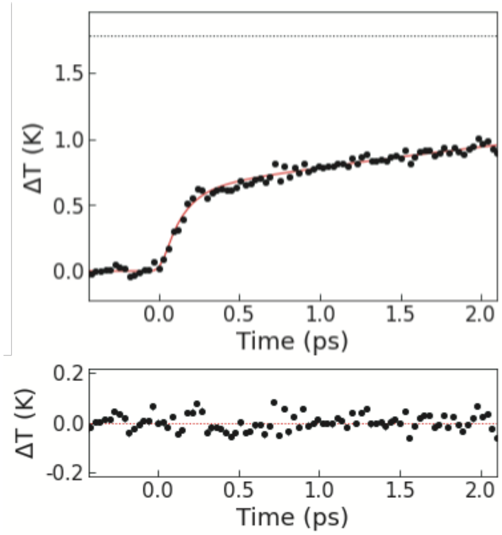

Figure S16. Temporal evolution of $\Delta T(t)$. (top) Black circles are the values quantified from the structural analysis. The black dotted line corresponds to $\Delta T(t)$ at 18.8 ps. The red line is the fitted curve. (bottom) Residuals of the kinetic fitting.

The temperature increase of the bulk solvent can be described by the sum of two exponential functions (see Note S3). The slow exponential time constant, τ_{exp1}, of 6.11 ps agrees well with the time constant assigned to vibrational cooling in a previous study using time-resolved X-ray absorption spectroscopy[17]. The temperature increase saturates at ~1.8 K. Given that the MLCT excited fraction (20.2%) decays into the relaxed T_1 state within < 20 ps, the excess energy released to the bulk solvent through vibrational cooling within 20 ps is estimated from the energy difference between the pump wavelength (550 nm) and the energy of the relaxed T_1 state with respect to the ground state (1.548 eV; the emission wavelength[21,22] of 700 nm minus the S_1–T_1 energy separation[23] of 1800 cm^{-1}), giving 2.286×10^{-20} J per liquid unit cell. Considering that the heat capacity[19] of acetonitrile is 63.53 $J\cdot mol^{-1}\cdot K^{-1}$, the expected temperature increase is calculated to be 1.13 K, which is 0.67 K lower than the measured value. This discrepancy implies the presence of an extra source of solvent heating, which is discussed below.

Firstly, we exclude the possibility of solvent heating via multiphoton absorption directly in the solvent, since a previous time-resolved XSS study[24] reported no temperature increase in neat

acetonitrile even when using a higher laser peak power than in the present study. Secondly, we consider the two-photon absorption by $[Cu(dmphen)_2]^+$. In a power titration scan (Figure. S17), $\Delta T(t)$ increases linearly up to 15.9 μJ, the laser power that was selected in most of the measurements. Simultaneous two-photon absorption gives a nonlinear behavior of $\Delta T(t)$ as a function of the laser power, and can, therefore, be excluded. Instead, the observed linear behavior indicates the possibility of sequential two-photon absorption, though a stepwise process, mediated by the excited state absorption (ESA). In this case, the second photon must be absorbed within the time duration of the pump pulse (< 50 fs) and the absorption cross section of the ESA should be non-negligible at the photoexcitation wavelength, *i.e.*, 550 nm. Indeed, according to the literature[14,25], transient absorption spectra of $[Cu(dmphen)_2]^+$ are characterized by a broad ESA band with a central wavelength of ~560 nm, which corresponds to the absorption of a phenanthroline radical anion (dmp·−), yielding higher-lying MLCT (S_n or T_n) states. Excitation of the ligand radical anion induced by a second photon preserves the Cu^{2+} (d^9) electronic configuration and, thus, gives a Cu Kα XES difference signal indistinguishable from that of the S_1 and T_1 MLCT states. The higher-lying MLCT states generated via ESA are expected to follow dynamics similar to the known relaxation after photoexcitation to the S_2 and S_3 states[11], *i.e.*, rapid nonradiative decay (internal conversion and intersystem crossing) to the S_1 and T_1 states, which is completed within ~50 fs. Hence, we conclude that the ESA process does not significantly affect the subsequent structural dynamics observed in this study. The difference between the calculated and measured $\Delta T(t)$ of the bulk solvent is rationalized by a ~3.7% fraction of molecules excited through ESA. This fraction represents an upper limit of the ESA branching, since other possible extra heat sources, *e.g.*, impulsive stimulated Raman scattering, are not taken into account.

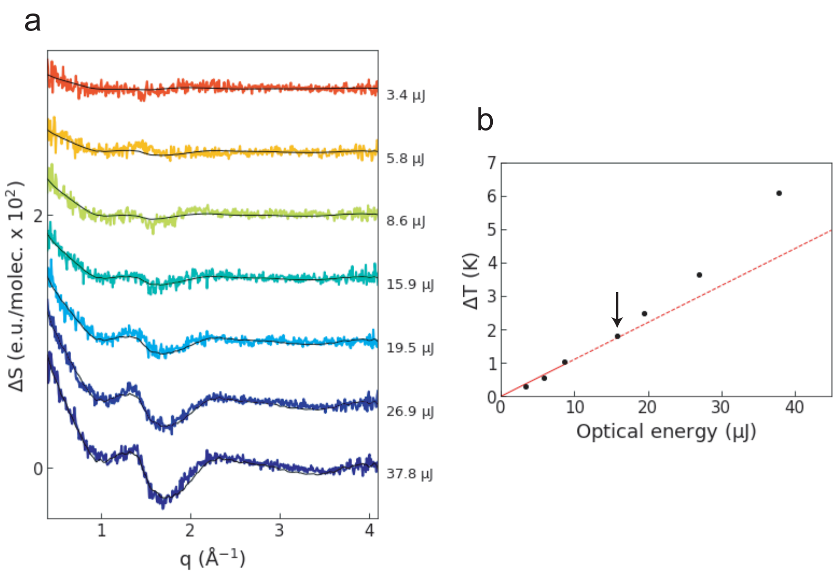

Figure S17. Optical power titration scan. (a) Colored lines are the difference scattering signals measured at 14.2 ps using different optical pulse energies. Black lines are the results of a structural analysis performed by constraining the $\Delta\theta$, Δr, and α parameters to the values obtained at 18.8 ps. (b) Black circles show ΔT quantified from the fitting. The red solid line corresponds to a linear fit in the range up to 8.6 μJ, while the red dashed line is an extrapolation to higher optical energies. The black arrow indicates the data point at 15.9 μJ.

The ESA process can also generate the oxidized species $[Cu(dmphen)_2]^{2+}$ and solvated electrons, similarly to what observed in $[Fe(bpy)_3]^{2+}$ and $[Ru(bpy)_3]^{2+}$ (bpy = 2,2'-bipyridine) aqueous solutions[26,27]. However, the Cu Kα XES difference spectra are sensitive to the generation of oxidized species, as supported by measurements of the time-resolved Cu Kα XES difference spectra at an optical pulse energy of 26.9 μJ (Figure S18). Indeed, the first RSV of an SVD of these spectra shows a prompt rise followed by a decay within ~2 ps, in contrast to the lack of a decay in the first RSV for the measurements with 15.9 μJ photoexcitation (Figure 2 of the main text). The features observed with 26.9 μJ excitation are interpreted as a fingerprint of the generation and ultrafast geminate recombination of oxidized species $[Cu(dmphen)_2]^{2+}$ and solvated electrons. Since these features are not observed in the XES spectra at the selected optical pulse energy of 15.9 μJ, contamination due to generation of oxidized species and solvated electrons can be excluded for the measurements presented and discussed in the main text.

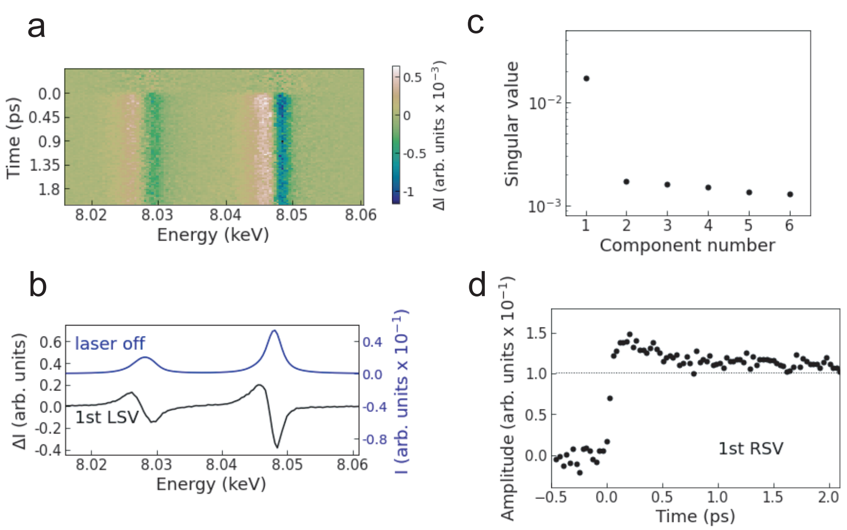

Figure S18. Time-resolved Cu Kα XES difference spectra measured at 26.9 µJ. (a) Transient Cu Kα XES spectra and (b)–(d) results of an SVD analysis. (b) 1st LSV (black line) and static spectrum (blue line). (c) Singular values of the 1st–6th components. (d) Black circles are the 1st RSV. The dotted line corresponds to the amplitude of the 1st RSV at 18.8 ps.

Note S6: SVD analysis of isotropic and anisotropic scattering data

SVD provides information on how many components are required to describe the measured data, albeit the extracted singular vectors do not necessarily correspond to physically meaningful signals. Figure S19 shows the isotropic ($\Delta S(q,t)$) and anisotropic ($\Delta S_2(q,t)$) X-ray scattering data and the results of SVD analyses. For both isotropic and anisotropic signals, there are four components exhibiting a clear temporal dependence, as deduced from the corresponding RSVs. This is evidence that the quantitative structural analysis using four time-dependent parameters does not overfit the measured data. In the anisotropic X-ray scattering, the first and second components dominate the signal. These components are associated with motions of acetonitrile molecules (the dynamics of libration and orientational diffusion), according to the time-resolved XSS study[24] on the optical Kerr effect.

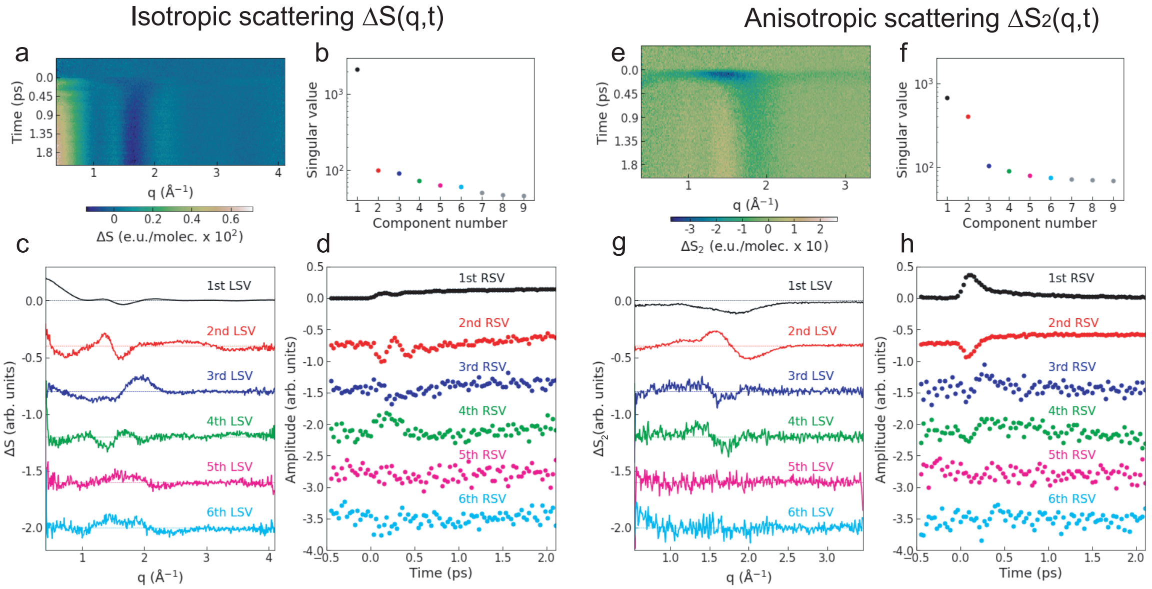

Figure S19. Isotropic and anisotropic scattering data and corresponding SVD analyses. (a–d) Isotropic signal and the SVD analysis. (e–f) Anisotropic signal and the SVD analysis. (a, e) Difference scattering signal as a function of time delay. (b, f) Singular values of the 1st–9th SVD components. (c, g) 1st–6th LSVs. (d, h) 1st–6th RSVs.

Figure S20 shows the normalized 1st RSVs of the SVD analyses of the time-resolved XSS and XES data. The onset of the initial rise in the 1st RSVs of isotropic $\Delta S(q,t)$ is slightly delayed (~30 fs) with respect to the 1st RSVs of anisotropic $\Delta S_2(q,t)$ and Cu Kα XES difference spectra. This means that the MLCT transition and the optical Kerr effect are instantaneously fast compared to the experimental IRF, while the solute structural changes start after the MLCT transition.

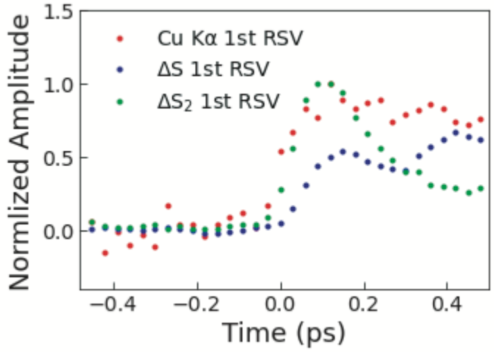

Figure S20. Normalized 1st RSVs of the SVD analyses of $\Delta S(q,t)$ (blue), $\Delta S_2(q,t)$ (green), and the time-resolved Cu Kα XES difference spectra (red).

Note S7: Vibrational analysis of excited state simulations

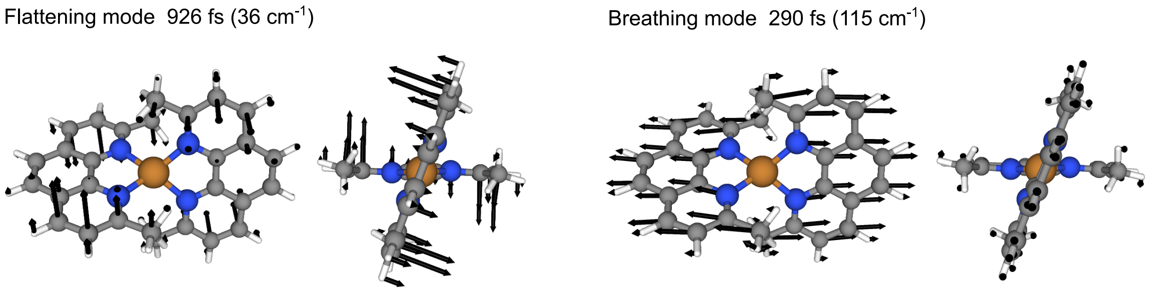

Figure S21. Normal modes from vibrational analysis in the S_1 excited state in vacuum. Displacement vectors of the flattening and breathing modes obtained from a vibrational analysis at the S_1 optimized geometry using time-independent variational density functional calculations with BLYP (ΔSCF approach employed in the QM/MM MD simulations[3]). The vibrational analysis is performed in the harmonic approximation using central finite difference calculation of the Hessian matrix as implemented in the Atomic Simulation Environment with analytical atomic forces converged to 10^{-4} eV/Å. The periods and frequencies of the modes are also indicated.

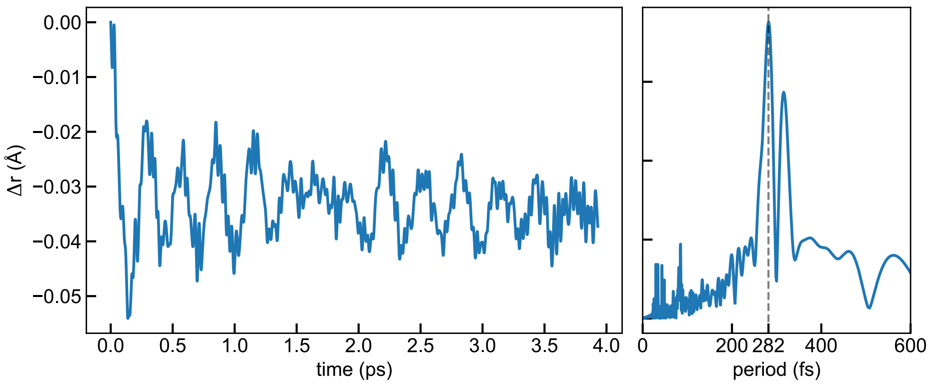

Figure S22. Evolution of the mean Cu–N distance from the QM/MM MD simulations in the S_1 excited state. (Left) The instantaneous change in mean Cu-N distance computed as average over the nonequilibrium excited state QM/MM MD trajectories. (Right) Fourier transform of the simulated instantaneous change in the mean Cu–N distance with a main peak at ~282 fs.

Supplementary References

1. T. Katayama, Y. Inubushi, Y. Obara, T. Sato, T. Togashi, K. Tono, T. Hatsui, T. Kameshima, A. Bhattacharya, Y. Ogi, N. Kurahashi, K. Misawa, T. Suzuki, and M. Yabashi, *Appl. Phys. Lett.*, 2013, **103**, 131105.

2. T. Katayama, S. Owada, T. Togashi, K. Ogawa, P. Karvinen, I. Vartiainen, A. Eronen, C. David, T. Sato, K. Nakajima, Y. Joti, H. Yumoto, H. Ohashi, and M. Yabashi, *Struct. Dyn.*, 2016, **3**, 034301.

3. G. Levi, E. Biasin, A. O. Dohn, and H. Jónsson, *Phys. Chem. Chem. Phys.*, 2020, **22**, 748–757.

4. J. N. Canongia Lopes, J. Deschamps, and A. A. H. Pádua, *J. Phys. Chem. B*, 2004, **108**, 2038–2047.

5. F. Hajdu, *Acta Cryst.*, 1972, **A28**, 250–252.

6. U. Lorenz, K. B. Møller, and N. E. Henriksen, *New J. Phys.*, 2010, **12**, 113002.

7. E. Biasin, T. B. van Driel, G. Levi, M. G. Laursen, A. O. Dohn, A. Moltke, P. Vester, F. B. K. Hansen, K. S. Kjaer, T. Harlang, R. Hartsock, M. Christensen, K. J. Gaffney, N. E. Henriksen, K. B. Møller, K. Haldrup, and M. M. Nielsen, *J. Synchrotron Radiat.*, 2018, **25**, 306–315.

8. J. R. Taylor, *An Introduction to Error Analysis.* (University Science Books, 1997).

9. K. Haldrup, M. Christensen, and M. M. Nielsen, *Acta Cryst. A*, 2010, **66**, 261–269.

10. K. Haldrup, M. Christensen, M. Cammarata, Q. Kong, M. Wulff, S. O. Mariager, K. Bechgaard, R. Feidenhans'l, N. Harrit, and M. M. Nielsen, *Angew. Chem. Int. Ed.*, 2009, **48**, 4180–4184.

11. M. Iwamura, S. Takeuchi, and T. Tahara, *J. Am. Chem. Soc.*, 2007, **129**, 5248–5256.

12. D. Felder, J. Nierengarten, F. Barigelletti, B. Ventura, and N. Armaroli, *J. Am. Chem. Soc.*, 2001, **123**, 6291–6299.

13. L. X. Chen, G. Jennings, T. Liu, D. J. Gosztola, J. P. Hessler, D. V. Scaltrito, and G. J. Meyer, *J. Am. Chem. Soc.*, 2002, **124**, 10861–10867.

14. L. X. Chen, G. B. Shaw, I. Novozhilova, T. Liu, G. Jennings, K. Attenkofer, G. J. Meyer, and P. Coppens, *J. Am. Chem. Soc.*, 2003, **125**, 7022–7034.

15. S. Smidstrup, A. Pedersen, K. Stokbro, and H. Jónsson, *J. Chem. Phys.*, 2014, **140**, 214106.

16. M. Iwamura, H. Watanabe, K. Ishii, S. Takeuchi, and T. Tahara, *J. Am. Chem. Soc.*, 2011, **133**, 7728–7736.

17. T. Katayama, T. Northey, W. Gawelda, C. J. Milne, G. Vankó, F. A. Lima, R. Bohinc, Z. Németh, S. Nozawa, T. Sato, D. Khakhulin, J. Szlachetko, T. Togashi, S. Owada, S. Adachi, C. Bressler, M. Yabashi, and T. J. Penfold, *Nat. Commun.*, 2019, **10**, 3606.

18. M. Cammarata, M. Lorenc, T. K. Kim, J. H. Lee, Q. Y. Kong, E. Pontecorvo, M. Lo Russo, G. Schiró, A. Cupane, M. Wulff, and H. Ihee, *J. Chem. Phys.*, 2006, **124**, 124504.

19. K. S. Kjær, T. B. van Driel, J. Kehres, K. Haldrup, D. Khakhulin, K. Bechgaard, M. Cammarata, M. Wulff, T. J. Sørensen, and M. M. Nielsen, *Phys. Chem. Chem. Phys.*, 2013, **15**, 15003–15016.

20. K. P. Burham and D. R. Anderson, *Model selection and multimodal inference: A practical information-theoretic approach* (Springer, ed. 2, 2002), ISBN 0-387-95364-7.

21. T. J. Penfold, S. Karlsson, G. Capano, F. A. Lima, J. Rittmann, M. Reinhard, M. H. Rittman-Frank, O. Braem, E. Baranoff, R. Abela, I. Tavernelli, U. Rothlisberger, C. J. Milne, and M. Chergui, *J. Phys. Chem. A*, 2013, **117**, 4591–4601.

22. M. W. Mara, K. A. Fransted, and L. X. Chen, *Coord. Chem. Rev.*, 2015, **282–283**, 2–18.

23. J. R. Kirchhoff, R. E. Gamache Jr., M. W. Blaskie, A. A. Del Paggio, R. K. Lengel, and D. R. McMillin, *Inorg. Chem.*, 1983, **22**, 2380–2384.

24. H. Ki, S. Choi, J. Kim, E. H. Choi, S. Lee, Y. Lee, K. Yoon, C. W. Ahn, D.-S. Ahn, J. H. Lee, J. Park, I. Eom, M. Kim, S. H. Chun, J. Kim, H. Ihee, and J. Kim, *J. Am. Chem. Soc.*, 2021, **143**, 14261–14273.

25. C. E. A. Palmer, D. R. McMillin, C. Kirmaier, and D. Holten, *Inorg. Chem.*, 1987, **26**, 3167–3170.

26. K. Haldrup, W. Gawelda, R. Abela, R. Alonso-Mori, U. Bergmann, A. Bordage, M. Cammarata, S. E. Canton, A. O. Dohn, T. B. van Driel, D. M. Fritz, A. Galler, P. Glatzel, T. Harlang, K. S. Kjær, H. T. Lemke, K. B. Møller, Z. Németh, M. Pápai, N. Sas, J. Uhlig, D. Zhu, G. Vankó, V. Sundström, M. M. Nielsen, and C. Bressler, *J. Phys. Chem. B*, 2016, **120**, 1158–1168.

27. A. N. Tarnovsky, W. Gawelda, M. Johnson, C. Bressler, and M. Chergui, *J. Phys. Chem. B*, 2006, **110**, 26497–26505.
